# Dimeric mimetic of BDNF loop 4 promotes survival of serum-deprived cell through TrkB-dependent apoptosis suppression

**DOI:** 10.1038/s41598-021-87435-0

**Published:** 2021-04-08

**Authors:** L. F. Zainullina, Yu. V. Vakhitova, A. Yu. Lusta, T. A. Gudasheva, S. B. Seredenin

**Affiliations:** Federal State Budgetary Institution “Research Zakusov Institute of Pharmacology”, 125315, Baltiyskaya str. 8, Moscow, Russia

**Keywords:** Biochemistry, Drug discovery, Neuroscience

## Abstract

Brain-derived neurotrophic factor (BDNF) is involved in the regulation of neuronal cell growth, differentiation, neuroprotection and synaptic plasticity. Although aberrant BDNF/TrkB signaling is implicated in several neurological, neurodegenerative and psychiatric disorders, neurotrophin-based therapy is challenging and is limited by improper pharmacokinetic properties of BDNF. Dimeric dipeptide compound GSB-106 (bis-(*N*-monosuccinyl-l-seryl-l-lysine) hexamethylenediamide) has earlier been designed to mimic the TrkB-interaction 4 loop of BDNF. It displayed protective effect in various cell-damaging models in vitro. Animal studies uncovered antidepressive and neuroprotective properties upon GSB-106 per os administration. Current study shows that GSB-106 acts similarly to BDNF, promoting survival of serum-deprived neuronal-like SH-SY5Y cells. 100 nmol concentration of GSB-106 provided maximum neurotrophic effect, which corresponds to about 37% of the maximum effect provided by BDNF. Protective properties of GSB-106 arise from its ability to counteract cell apoptosis via activation of TrkB-dependent pro-survival mechanisms, including inactivation of pro-apoptotic BAD protein and suppression of caspases 9 and 3/7. Thus, our study has characterized neurotrophic activity of small dimeric compound GSB-106, which mimics certain biological functions of BDNF and neurotrophin-specific protective mechanisms. GSB-106 also displays similarities to some known low weight peptide and non-peptide TrkB ligands.

## Introduction

Brain-derived neurotrophic factor (BDNF) is a secreted protein that serves neuronal survival, cell differentiation and synaptic plasticity through interactions with tropomyosin-related kinase-B receptor (TrkB) and tumor necrosis factor receptor superfamily member 16 (p75^NTR^)^1^. BDNF binding to TrkB evokes receptor dimerization and initial phosphorylation of tyrosine residues within the autoregulatory loop of the kinase domain (human TrkB Tyr^706/707^) followed by autophosphorylation of cytoplasmic conserved tyrosine residues (human TrkB Tyr^515^, Tyr^816^)^2^. Phosphorylated TrkB Tyr^516^ through the interaction with scaffold protein Shc (Src homologous and collagen-like) mediates the activation of PI3K (Phosphatidylinositol 3-kinase) via Grb2 (Growth factor receptor-bound protein 2) and Gab1 (Grb2-associated binder-1) proteins, which in turn leads to Akt (Rac-alpha serine/threonine-protein kinase) activation, promotion of Akt-dependent neurotrophin survival effects and increased protein translation (regulated by mTOR-p70S6 kinase). Additionally, a Shc binding site at Tyr^515^ initiates Ras/Raf/MEK/Erk cascade responsible for the neuronal differentiation, neuronal outgrowth and synaptic plasticity via recruitment of Grb2 and SOS (Son of sevenless) proteins^1^. Phosphorylation of Tyr^816^ leads to PLC-γ1 (phospholipase Cγ) activation, which through DAG (diacylglycerol) and IP_3_ (inositol tris–phosphate) results in activation of Ca^2+^—and protein kinase C-regulated pathways that facilitate synaptic plasticity^1^.

In addition to binding with TrkB BDNF also engages p75^NTR^ and this interaction mediate distinct outcomes, depending on the cellular milieu. In brief, when TrkB and p75^NTR^ co-express in the same cells, p75^NTR^ enhances neurotrophin binding to TrkB, augmenting both ligand affinity and selectivity, hence, reinforcing the TrkB-mediated trophic response via up-regulation of PI3K/Akt and/or IRAK/NF-κB signaling^3^. Moreover, in specific context, p75^NTR^ can activate TrkB-independent pro-survival signals, which involve activation of transcription factor NF-κB and PI3K/Akt pathway^4,5^. Degenerative pathways would dominate, promoting cell death upon neurotrophin binding to p75^NTR^ through activation of pro-apoptotic JNK-p53-Bax pathway or suppressing Ras/PI3K/Akt activation when conditions of either lacking or deficient Trk signaling or increased p75^NTR^ expression occur^6^. Implication of p75^NTR^ in modulation of myelin formation, neurite outgrowth and pro-neurotrophin-mediated signaling also widely discussed^7^.

Deficiency of neurotrophin and/or disturbances in BDNF/TrkB/p75^NTR^—dependent signaling contributes to pathogenesis and progression of numerous neurological and psychiatric disorders, denoting protective and regenerative properties of BDNF^8^. Given the important role that BDNF plays in human pathology, intensive efforts have been made to develop strategies for therapeutic applications of neurotrophins or their functional analogs. Clinical trials showed that exogenous BDNF had limited success as therapeutic, mainly due to suboptimal pharmacokinetic properties and side effects. Nevertheless, encouraging pre-clinical data have emerged for certain substances with favorable pharmacokinetic profiles, targeting the BDNF/TrkB-associated pathway to restore or balance the altered neurotrophin signaling^9^. A vast number of agents targeting neurotrophin receptors with different action mechanisms have been developed and studied in details in vitro and in various animal disorders models recently^10–14^.

**Figure 1:**
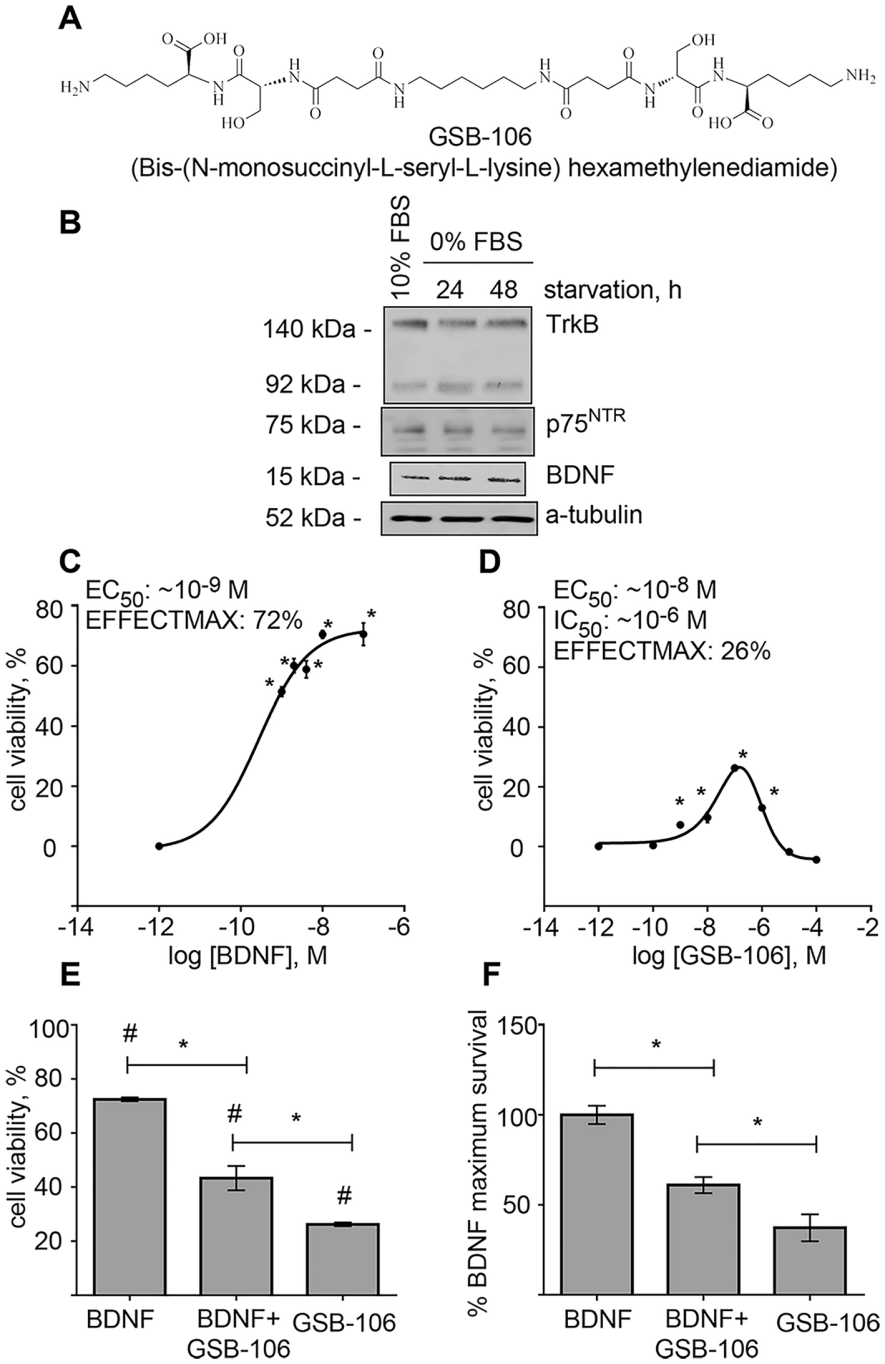
GSB-106 promotes survival of serum-deprived SH-SY5Y cells. (**A**) GSB-106 chemical structure. (**B**) BDNF, TrkB and p75^NRT^ proteins expression in serum starved SH-SY5Y cells. Cells were incubated in serum-free culture medium (“0% FBS”) for 24 and 48 h. After incubation, cells were collected, and protein extracts were subjected to polyacrylamide gel electrophoresis and transferred for Western blotting. Blots were probed with anti-BDNF, anti-TrkB, anti-p75^NTR^ antibodies and anti-a-tubulin antibody. The figure shows data from one independent experiment (n = 3; the original blots are shown in the Supplementary Information file). (**C**,**D**) Dose–response survival curves of SH-SY5Y cells (2 × 10^5^/well) treated with BDNF (**C**) or GSB-106 (**D**) for 48 h in serum-free condition. Cell viability was measured by MTT metabolism. Cell viability was normalized to viability in control group (“0% FBS”) shown as “10^–12^ M” (*p < 0.05; n = 7; one-way ANOVA with Newman-Keul’s post-hoc test). (**E**) Survival of cells (2 × 10^5^/well) incubated with BDNF (100 nmol), GSB-106 alone (100 nmol) and GSB-106 + BDNF (100 nmol for BDNF and GSB-106) in serum-free DMEM for 48 h. Cell viability was normalized to viability in control group (“0% FBS”) (*p < 0.05; ^#^p < 0.05 in relation to control cells (“0% FBS”); n = 7; Wilcoxon *t*-test). (**F**) Survival of cells (2 × 10^5^/well) incubated with BDNF (100 nM), GSB-106 alone (100 nmol) and GSB-106 + BDNF (100 nmol for BDNF and GSB-106) in serum-free DMEM for 48 h. Cell viability was normalized to viability in group with BDNF alone (shown as 100% on Y-axis) (*p < 0.05; n = 7; Wilcoxon *t*-test). Hereinafter (overall) the data is expressed as means ± S.E.M.

Orally available dimeric dipeptide GSB-106 (Fig. 1A; bis-(*N*-monosuccinyl-l-seryl-l-lysine) hexamethylenediamide) has earlier been designed based on BDNF loop 4 β-turn amino acid sequence -Asp^93^-**Ser**^**94**^**-Lys**^**95**^-Lys^96^-, where -**Ser**^**94**^**-Lys**^**95**^-residues were retained, the upstream residue -Asp^93^- was replaced by its bioisostere, a succinic acid residue, and -Lys^96^- residue was substituted by the amide group^15,16^. Previous studies demonstrated that GSB-106 exerted a trophic effect, up-regulated the TrkB phosphorylation as well as the downstream PI3K/Akt, MAPK/Erk and PLCγ signaling pathways in vitro^17–19^. It has been shown earlier that GSB-106 over concentration range of 10 nmol -1 µmol rescues HT-22 cells from oxidative stress and glutamate-induced cell death and protects SH-SY5Y cells against 6-hydroxydopamine damage^20^. The substance was found to possess the favorable pharmacokinetic properties, such as proteolytic stability, blood–brain barrier penetration, retention in rat blood plasma for 4 h upon single *per os* administration; T_1/2_ for GSB-106 is 0.7 h^21^. GSB-106 has been shown to counteract depression-like behavior in a number of rodent tests and in animal models of depression^15,22–24^ and exhibited pronounced neuroprotective activity in animal ischemic stroke model upon *per os* administration^25,26^.

Our research aimed to characterize the activity profile of GSB-106 as a small molecule TrkB ligand and to find out whether GSB-106 could alleviate the cell death triggered by serum-deprivation via mechanisms similar to those of BDNF.

## Results

### GSB-106 provides neuroprotection from serum withdrawal-induced cell apoptosis

Neurotrophic activity of GSB-106 was studied in undifferentiated SH-SY5Y neuroblastoma cells, cultured in the serum free conditions to exclude the presence of exogenous trophic factors. Although controversial reports on the expression of TrkB in undifferentiated SH-SY5Y cells in culture exist^27^, we used immunoblotting to show that these cells indeed express TrkB receptor proteins during cultivation in complete serum (Fig. 1B). Expression of TrkB, p75^NTR^ receptors and small amount of BDNF protein has also been confirmed in serum-deprived cells (Fig. 1B).

Based on the observation that SH-SY5Y cells express TrkB and p75^NTR^ receptors, we investigated whether BDNF and its low molecular weight mimetic protect SH-SY5Y cells from serum withdrawal. Recombinant human BDNF was used as internal standard. 48 h treatment with GSB-106 alone (0.1 nmol–100 µmol) produced a bell-shaped concentration–response curve with a maximum survival effect (E_max_) of 26.25 ± 0.67% at 100 nmol compared to control group (0% FBS; p < 0.05; one-way ANOVA with Newman–Keul’s post-hoc test); EC_50_ value is 10 nmol. Higher concentrations of the compound tended to inhibit the cell viability (IC_50_ of 1 µmol) (Fig. 1D). Neurotrophic activity studies with BDNF (1 nmol–1 µmol) demonstrated a sigmoidal curve with a maximum survival BDNF effect (E_max_) of 72.5 ± 0.7% at 100 nmol compared to control group (0% FBS; p < 0.01; one-way ANOVA with Newman–Keul’s post-hoc test); EC_50_ value is 1 nmol (Fig. 1C). Statistically significant differences were established when comparing the values of the maximum effects of BDNF and GSB-106 (p < 0.05; Wilcoxon *t*-test). Thus, GSB-106 promoted cell survival with lower potency compared to BDNF, as evident from the half-maximum concentrations of BDNF and GSB-106, which differ by 1 order: EC_50_ of 1 nmol for BDNF versus EC_50_ 10 nmol for GSB-106. To be noted, maximum neurotrophic effect was observed at 100 nmol concentrations for both BDNF and GSB-106, despite the clear differences in their efficiency (E_max_ of 72.5 ± 0.7% for BDNF versus E_max_ of 26.25 ± 0.67% for GSB-106). Obtained data indicate that survival of serum-deprived cells is dependent on the intrinsic trophic activity of GSB-106 solely, although this effect was less pronounced compared to the native neurotrophin. 100 nmol concentration of GSB-106 provided maximum neurotrophic effect, which corresponds to about 37% of the maximum effect provided by BDNF (p < 0.05; Wilcoxon *t*-test; Fig. 1F).

An increase of serum-depleted SH-SY5Y cells survival by 26.25 ± 0.67%, comparing with control group (0% FBS; p < 0.05; Wilcoxon *t*-test; Fig. 1E) was observed when GSB-106 (100 nmol) was added alone. Co-addition of GSB-106 with BDNF at concentrations, inducing maximum survival effect (100 nmol for each compound), resulted in 43.33 ± 4.48% enhancement of trophic response compared with control group (0% FBS; p < 0.05; Wilcoxon *t*-test; Fig. 1E), that is 60.98 ± 4.5% of BDNF maximum effect (p < 0.05; Wilcoxon *t*-test; Fig. 1F). It is worth noting that co-incubation with GSB-106 and BDNF raised pro-survival activity of dimeric dipeptide by 17.08 ± 1.6% (p < 0.05; Wilcoxon *t*-test; Fig. 1E), supposing that GSB-106 might act additively (or cooperatively) with BDNF. Competition analysis revealed that GSB-106 in the presence of saturated concentration of BDNF inhibited the maximum effect of BDNF by 39.02 ± 1.8% (p < 0.05; Wilcoxon *t*-test; Fig. 1F), suggesting that GSB-106 could function as partial agonist at concentration evoking maximum response.

Serum withdrawal commonly triggers cells apoptosis and represents a relevant model for studying the underlying mechanisms of neuroprotection arised from neurotrophic support^28^. We further examined in more depth, whether apoptosis could be downregulated by GSB-106 to elucidate the survival-promoting activity of compound in serum-deprived SH-SY5Y cells. Annexin V/SYTOX staining followed by flow cytometry has been used to detect apoptotic events. This approach allows distinguishing early apoptotic cells and late apoptotic/necrotic cells.

Upon 72-h serum starvation (0% FBS) percentage of early and late apoptotic cells has remarkably increased compared to that of cells cultured in complete serum media (10% FBS) as represented in Table 1. Treatment of serum-deprived SH-SY5Y cells with GSB-106 at concentrations of 10 nmol to 1 µmol alleviated the levels of early and late apoptosis to the nearly same extent as that of BDNF at concentration of ~ 1 nmol. Notably, an increase in both early and late apoptotic cells was shown for GSB-106 at concentrations of 10 µmol to 100 µmol, which is consistent with the data on cell viability (Fig. 1D). Taken together, these data indicates, that GSB-106 protects SH-SY5Y cells from serum withdrawal initiated apoptosis in a concentration-dependent manner.

**Table 1:** GSB-106 attenuates the apoptosis of serum withdrawn SH-SY5Y cells.

Groups	Apoptosis, % of cells		Necrosis
	Early	Late	
10% FBS	0.5 ± 0.01	1.8 ± 0.1	2.75 ± 0.5
0% FBS	4.32 ± 0.2	7.08 ± 0.09	2.92 ± 0.4
BDNF, ~ 1 nmol	3.85 ± 0.1*	5.9 ± 0.1*	2.74 ± 0.5
**GSB-106**			
10 nmol	3.68 ± 0.1*	5.85 ± 0.3*	3.15 ± 0.2
100 nmol	3.65 ± 0.09*	6.2 ± 0.3*	2.42 ± 0.6
1 µmol	3.9 ± 0.3	5.1 ± 0.2*	2.82 ± 0.4
10 µmol	5.2 ± 0.2	8.91 ± 0.1	2.81 ± 0.5
100 µmol	5.67 ± 0.1	8.18 ± 0.3	2.44 ± 0.3

SH-SY5Y cells were cultured in serum-free culture medium (0% FBS) and treated with BDNF.

(~ 1 nmol) or GSB-106 (10 nmol to 100 µmol) for 72 h. After incubation cells were collected and stained with Metabolic Activity Dead Cell Apoptosis Kit with C12 Resazurin, Annexin V APC, and SYTOX Green according to the manufacturer's recommendations. The stages of apoptosis were determined by staining: early—Annexin V^+^/SYTO Green^−^; late—Annexin V^+^/SYTO Green^+^; necrosis—Annexin V^−^/SYTO Green^+^. The data is presented as the mean ± S.E.M. of percentage of total cell population (n = 3; *p < 0.05 compared with the “0% FBS” group; Wilcoxon *t*-test).

### GSB-106 mediates neuroprotection through Trk signaling

**Figure 2:**
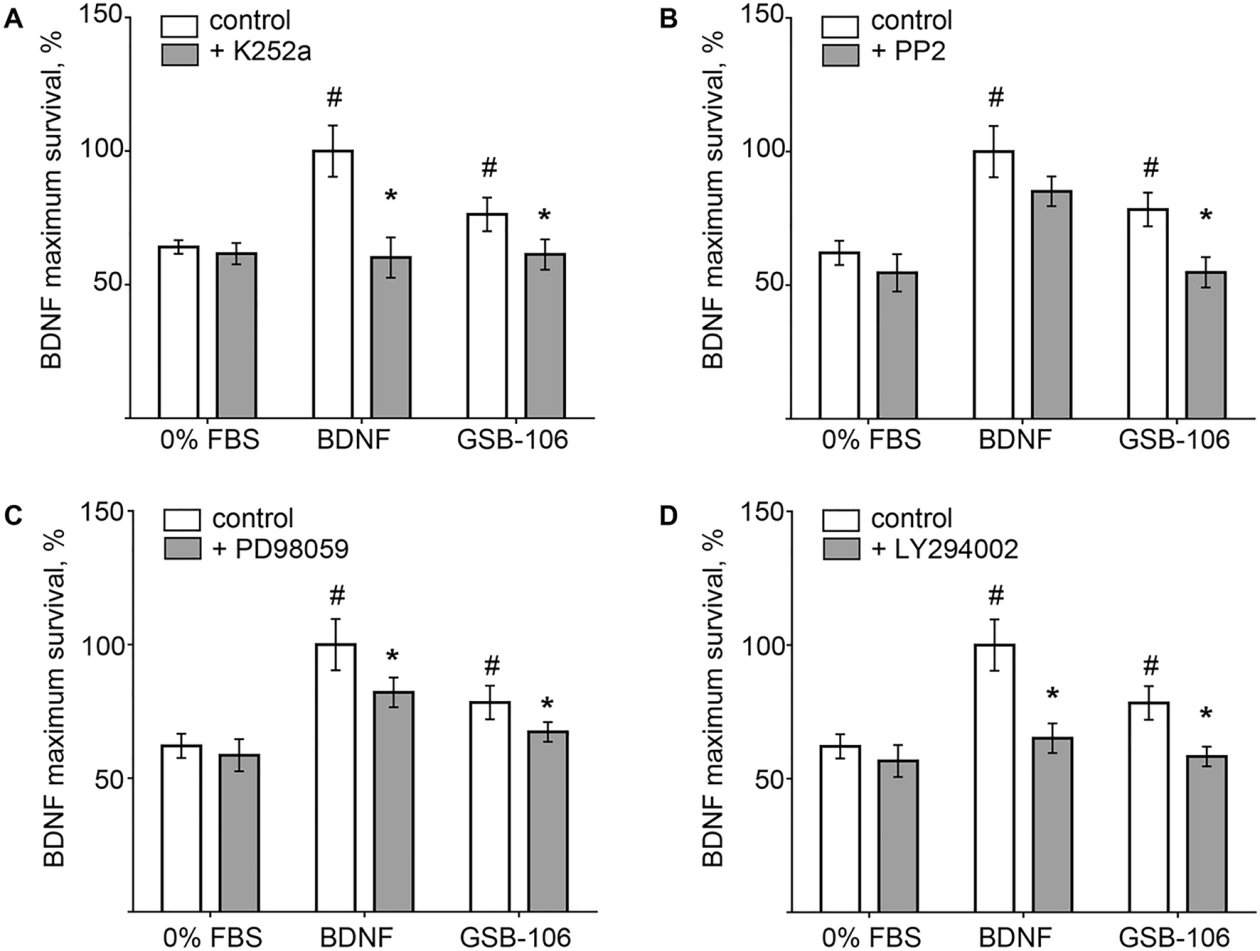
GSB-106 promotes survival of serum-deprived SH-SY5Y cells through TrkB and downstream signaling pathways. (**A**) Survival analysis of serum-deprived SH-SY5Y cells (2 × 10^5^/well) incubated with BDNF (1 nmol), GSB-106 (100 nmol) in the presence of K252a (500 nmol) for 48 h. Cell viability was normalized to viability in BDNF group (n = 5; ^#^p < 0.05 in relation to 0% FBS-control; *p < 0.05; in relation to agonist without inhibitor; Wilcoxon *t*-test). (**B**) Survival analysis of serum-deprived SH-SY5Y cells (2 × 10^5^/well) incubated with BDNF (1 nmol), GSB-106 (100 nmol) in the presence of PP2 (100 μmol) for 48 h. Cell viability was normalized to viability in BDNF group (n = 5; ^#^p < 0.05 in relation to 0% FBS-control; *p < 0.05; in relation to agonist without inhibitor; Wilcoxon *t*-test). (**C**) Survival analysis of serum-deprived SH-SY5Y cells (2 × 10^5^/well) incubated with BDNF (1 nmol), GSB-106 (100 nmol) in the presence of PD98059 (50 μmol) for 48 h. Cell viability was normalized to viability in BDNF group (n = 5; ^#^p < 0.05 in relation to 0% FBS-control; *p < 0.05; in relation to agonist without inhibitor; Wilcoxon *t*-test). (**D**) Survival analysis of serum-deprived SH-SY5Y cells (2 × 10^5^/well) incubated with BDNF (1 nmol), GSB-106 (100 nmol) in the presence of LY294002 (1 μmol) for 48 h. Cell viability was normalized to viability in BDNF group (n = 5; ^#^p < 0.05 in relation to 0% FBS-control; *p < 0.05; in relation to agonist without inhibitor; Wilcoxon *t*-test).

Survival responses in the presence of the kinase inhibitor K252a or the MEK1 (PD98059) and the PI3K (LY294002) inhibitors were evaluated to determine whether GSB-106 ability to support viability of serum-deprived cells is dependent on TrkB receptors activation and MAPK/Erk and PI3K/Akt pathways. K252a decreased BDNF neurotrophic activity by 40 ± 2.5% (p < 0.05; Wilcoxon *t*-test) and decreased GSB-106 activity by 15 ± 4.9% (p < 0.05; Wilcoxon *t*-test), as shown in Fig. 2A. These findings demonstrate that compound’s activity partially depends on TrkB kinase activation and could suggest also non-TrkB mediated effects of GSB-106. Indeed, Src kinases inhibitor PP2 (Fig. 2B) reduced the BDNF-stimulated cell viability by 15 ± 5.5% (p < 0.05; Wilcoxon *t*-test), whereas GSB-106-stimulated cell survival was inhibited by 23.5 ± 4.9% (p < 0.05; Wilcoxon *t*-test), intimating the plausible contribution of Src kinase-dependent cell survival mechanisms. Applying PD98059 (Fig. 2C) to serum-free cells blocked the survival promoted by BDNF or GSB-106 by 18 ± 5.5% (p < 0.05; Wilcoxon *t*-test) and by 14 ± 4.9% (p < 0.05; Wilcoxon *t*-test) respectively. LY294002 (Fig. 2D) inhibited BDNF-stimulated cell viability by 35 ± 6.8% (p < 0.05; Wilcoxon *t*-test) and GSB-106-supported survival by 20 ± 7.9% (p < 0.05; Wilcoxon *t*-test). Notably, PD98059 affected BDNF- or GSB-106-promoted survival to a lesser extent compared to LY294002, which is consistent with previous observations^29^. Although these inhibitors are not completely specific, obtained data suggests dependence of GSB-106-promoted survival on TrkB activation/Src-regulated transactivation and downstream MAPK/Erk, PI3K/Akt signaling.

**Figure 3:**
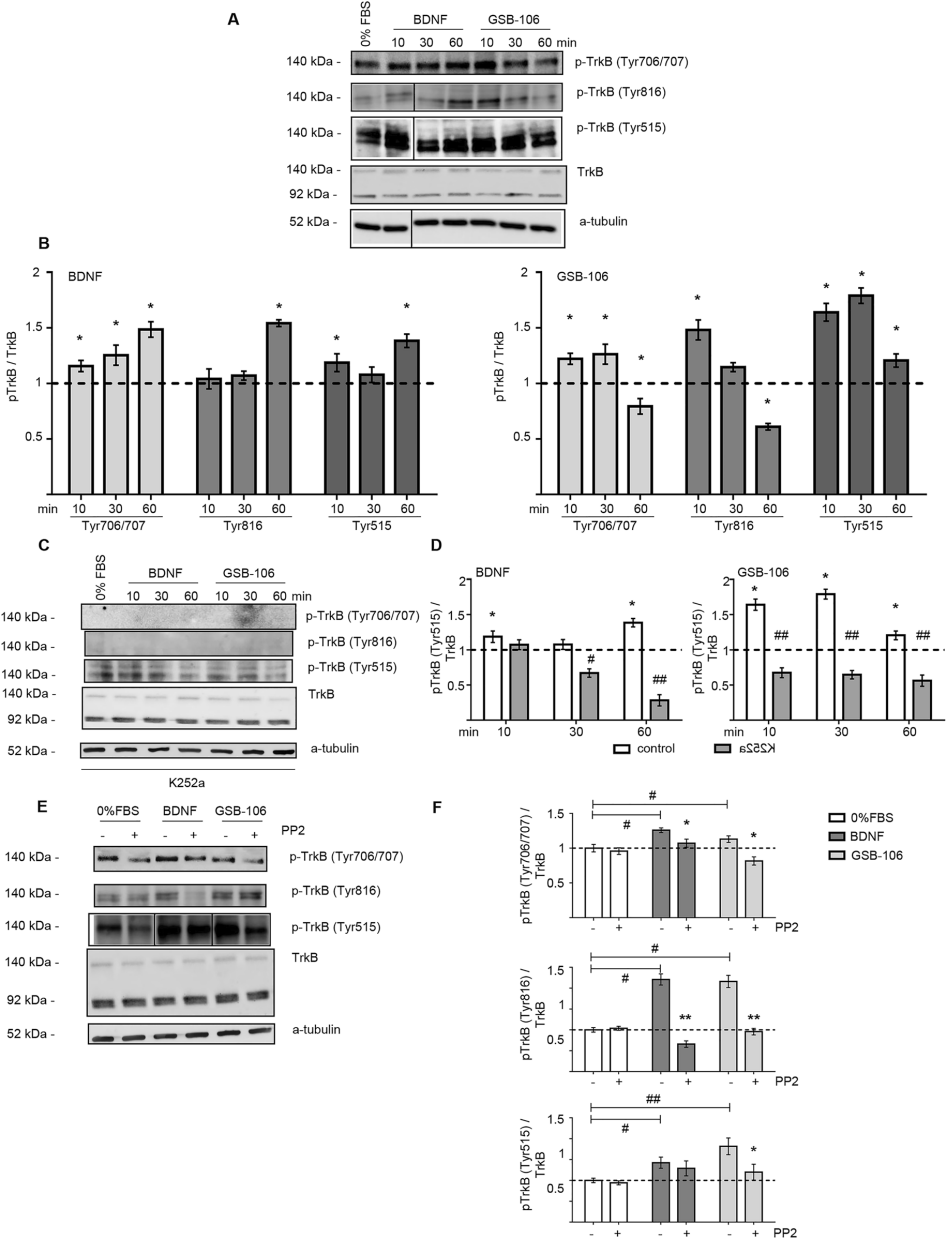
The neuroprotective effect of GSB-106 is mediated through the activation of TrkB. (**A**) SH-SY5Y cells were cultured in serum-free culture medium (“0% FBS” for 24 h) with or without BDNF (28.6 ng/ml, ~ 1 nmol) or GSB-106 (100 nmol). After incubation with BDNF or GSB-106 for 10, 30, 180 min cells were collected, and protein extracts were subjected to polyacrylamide gel electrophoresis and transferred for Western blotting. Blots were probed with anti-phosphorylated pTrkB (Tyr^706/707^), anti-phosphorylated pTrkB (Tyr^816^), anti-phosphorylated pTrkB (Tyr^515^), anti-TrkB antibody and anti-a-tubulin antibody. The figure shows data from one independent experiment (n = 3; separation bars indicate non-contiguous lanes on the same image acquisition (the original blots are shown in the Supplementary Information file). (**B**) The ratio of signal derived from phosphorylated TrkB over TrkB bands was calculated (n = 3; *p < 0.05 in relation to corresponding “0% FBS”, taken for 1 unit; Wilcoxon *t*-test). (**C**) SH-SY5Y cells in serum-free medium (“0% FBS”) with or without BDNF (~ 1 nmol) or GSB-106 (100 nmol) were also pre incubated in the presence or absence of K252a (500 nmol, 60 min). Blots were first probed with anti-phosphorylated antibodies and then reprobed with anti-TrkB antibody, anti-a-tubulin antibody. The figure shows data from one independent experiment (n = 3; the original blots are shown in the Supplementary Information file). (**D**) The ratio of signal derived from phosphorylated TrkB (Tyr^515^) over TrkB bands was calculated (n = 3; *p < 0.05 in relation to corresponding “0% FBS” (dashed line); ^#^p < 0.05, ^##^p < 0.01 in relation to corresponding groups without K252a; Wilcoxon *t*-test). (**E**) SH-SY5Y cells in serum-free culture medium (“0% FBS”) with or without BDNF (~ 1 nmol) or GSB-106 (100 nmol) were also pre incubated with or without PP2 (100 µmol, 30 min). Blots were probed with anti-phosphorylated pTrkB (Tyr^706/707^), anti-phosphorylated pTrkB (Tyr^816^) or anti-phosphorylated pTrkB (Tyr^515^) antibodies and anti-TrkB antibody, anti-a-tubulin antibody. The figure shows data from one independent experiment (n = 3; the original blots are shown in the Supplementary Information file). (**F**) The ratio of signal derived from phosphorylated TrkB over TrkB bands was calculated (n = 3; ^#^p < 0.05, ^##^p < 0.01 in relation to corresponding “0% FBS”; *p < 0.05, **p < 0.01 in relation to corresponding “- PP2”; Wilcoxon *t*-test).

Immunoblotting of TrkB with antibodies to specific phospho tyrosines has been performed to ascertain whether TrkB phosphorylation is induced by GSB-106. Figures 3A,B show that GSB-106 induced the transient and similar to BDNF moderate TrkB Tyr^706/707^ phosphorylation within 10 min, followed by a decrease at 60 min, whilst BDNF caused substantial Tyr^706/707^ phosphorylation at 60 min. The opposite time-dependent activation of Tyr^816^ was also observed. Maximum of Tyr^816^ phosphorylation (48.1 ± 9%; p < 0.05; Wilcoxon *t*-test) by GSB-106 has been detected at 10 min, and pick of BDNF-triggered activation (54.2 ± 2%; p < 0.05; Wilcoxon *t*-test) was observed at 60 min. Tyr^515^ phosphorylation was affected significantly more by GSB-106, than by BDNF. Particularly, the enhanced level of Tyr^515^ activation appeared as early as 10 min (64.0 ± 8%; p < 0.05; Wilcoxon *t*-test) and maintained for up to 60 min, although BDNF caused an increase in phosphorylation at this site only after 60 min (38.4 ± 6%; p < 0.05; Wilcoxon *t*-test).

BDNF and GSB-106 effects on TrkB Tyr^706/707^ and Tyr^816^ phosphorylation were nearly completely suppressed by K252a (Fig. 3C). However, Tyr^515^ phosphorylation, induced by BDNF was inhibited by 32.8 ± 6% at 30 min and by 71.7 ± 8% at 60 min (p < 0.05; Wilcoxon *t*-test), whereas cells incubation with GSB-106 resulted in 43.7 ± 8% (p < 0.01; Wilcoxon *t*-test) sustained inhibition at 60 min upon K252a treatment (Fig. 3C,D). Together, these findings suggested, that effects of GSB-106 and, at lesser extent, BDNF on phosphorylation of Tyr^515^ could be mediated not only by TrkB, but also by mechanisms, which act downstream of TrkB. K252a is a potent Trk inhibitor, capable to activate Akt and Erk kinases via Src-dependent pathway, thus supporting cell survival in serum withdrawn cells^30^. Currently, several TrkB-independent mechanisms described insensitive to K252a inhibition, including Src-dependent transactivation of neurotrophin receptor and maintaining of calcium signaling by truncated TrkB receptor isoforms^31^.

TrkB receptor is capable of autophosphorylation and activation of downstream cascades independent on BDNF through the transactivation mechanisms, which may involve GPCRs and are mediated by Src family kinases (SFK)^32^. To find out whether GSB-106 might affect TrkB signaling via Src-dependent transactivation mechanisms, the effect of Src kinases inhibitor PP2 on site-specific TrkB phosphorylation (Tyr^706/707^, Tyr^515^, and Tyr^816^) was determined. PP2 alone (100 µmol) did not affect the phosphorylation of studied tyrosines in control serum-deprived cells (Fig. 3E). Addition of PP2 resulted in a slightly reduced BDNF-induced phosphorylation of Tyr^706/707^ and Tyr^515^, while a more significant inhibition of Tyr^816^ phosphorylation was found (Fig. 3E,F). These data are consistent with observations, that chemically diverse inhibitors of Src family kinases suppressed the BDNF-evoked Tyr^706/707^, Tyr^515^, and Tyr^816^ TrkB phosphorylation, implicating SFKs as regulators of TrkB activation by BDNF^33^. Pre-incubation of cells with PP2 led to a modest reduction of GSB-106-induced Tyr^706/707^ phosphorylation compared to that of Tyr^515^, and more apparent decline of Tyr^816^ phosphorylation (p < 0.05, p < 0.01; Wilcoxon *t*-test; Fig. 3E,F). Remarkably, PP2 altered the GSB-106-elicited Tyr^706/707^ and Tyr^515^ phosphorylation more profoundly than that induced by BDNF, whereas the decrease of Tyr^816^ phosphorylation was less striking in case of GSB-106. Altogether, our data shows that SFK inhibitor PP2 preferentially reduced both BDNF- and GSB-106-mediated phosphorylation of Tyr^816^ compared to Tyr^706/707^ and Tyr^515^ under given experimental conditions. Collectively, our findings suggest that TrkB activation by GSB-106 was, at least, partially dependent on Src kinases-implicated TrkB transactivation mechanisms.

**Figure 4:**
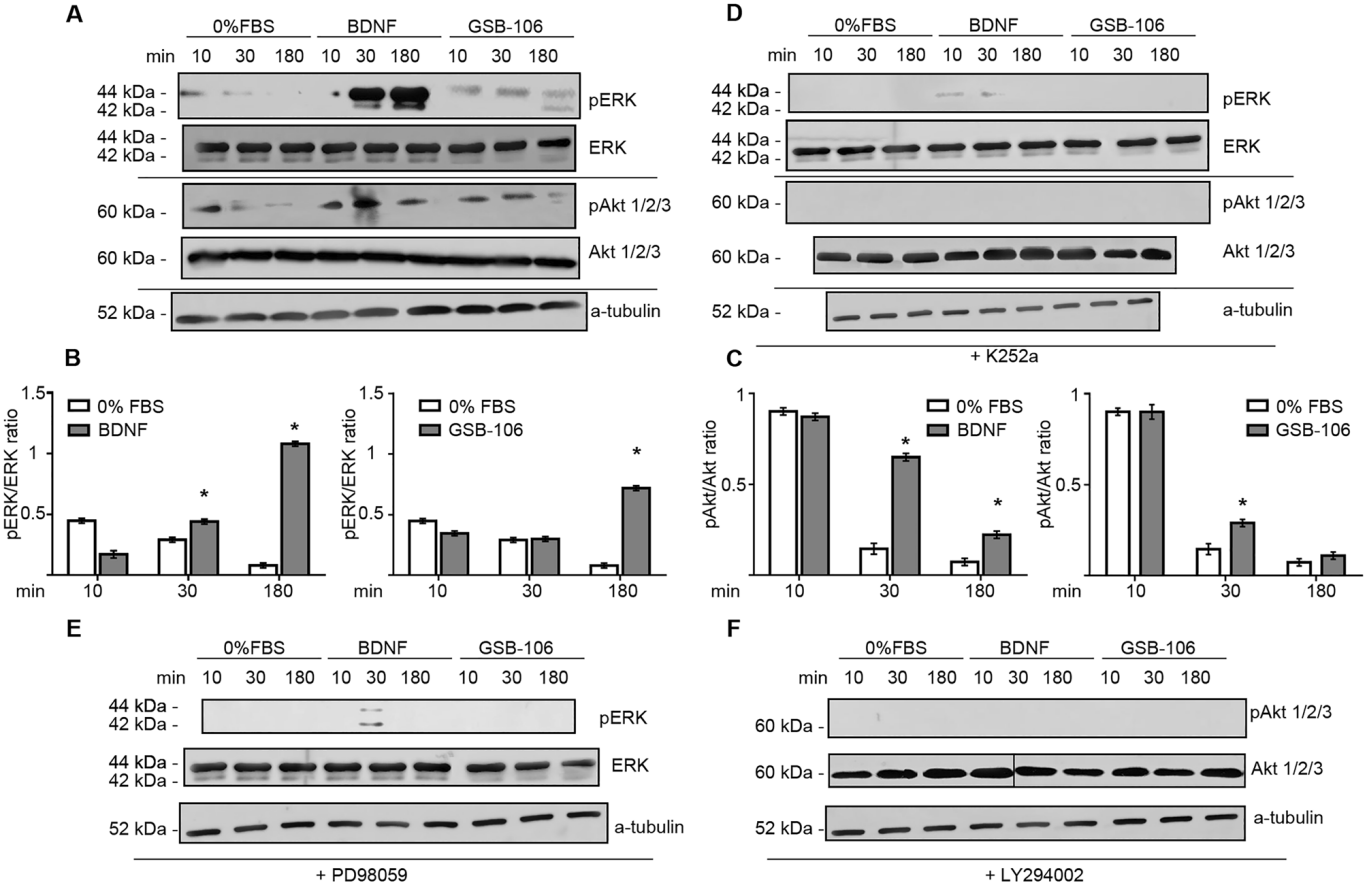
The neuroprotective effect of GSB-106 is mediated through the activation of MAPK/Erk and PI3K/Akt pathways. (**A**) SH-SY5Y cells were incubated in serum-free culture medium (“0% FBS”) with or without BDNF (28.6 ng/ml, ~ 1 nmol) or GSB-106 (100 nmol) for 10, 30 or 180 min. After incubation, cells were collected, and protein extracts were subjected to polyacrylamide gel electrophoresis and transferred for Western blotting. Blots were first probed with anti-phosphorylated antibodies and then reprobed with anti-Erk or anti-pan-Akt antibodies, anti-a-tubulin antibody. The figure shows data from one independent experiment (n = 3; the original blots are shown in the Supplementary Information file). (**B**) The ratio of signal derived from phosphorylated Erk over Erk bands was calculated (n = 3; *p < 0.05 in relation to corresponding “0% FBS”; Wilcoxon *t*-test). (**C**) The ratio of signal derived from phosphorylated Akt over Akt bands was calculated (n = 3; *p < 0.05 in relation to corresponding “0% FBS”; Wilcoxon *t*-test). (**D**) SH-SY5Y cells in serum-free culture medium (“0% FBS”) with or without BDNF (~ 1 nmol) or GSB-106 (100 nmol) were also pre incubated in the presence or absence of K252a (500 nmol, 60 min). Blots were first probed with anti-phosphorylated antibodies and then reprobed with anti-Erk or anti-pan-Akt antibodies, anti-a-tubulin antibody. The figure shows data from one independent experiment (n = 3; the original blots are shown in the Supplementary Information file). (**E**) SH-SY5Y cells in serum-free culture medium (“0% FBS”) with or without BDNF (~ 1 nmol) or GSB-106 (100 nmol) were also pre incubated with or without PD98059 (50 µmol, 60 min). Blots were first probed with anti-phosphorylated Erk (pErk) antibody and then reprobed with anti-Erk antibody, anti-a-tubulin antibody. The figure shows data from one independent experiment (n = 3; the original blots are shown in the Supplementary Information file). (**F**) SH-SY5Y cells in serum-free culture medium (“0% FBS”) with or without BDNF (~ 1 nmol) or GSB-106 (100 nmol) were also pre incubated with or without LY290042 (50 µmol, 60 min). Blots were first probed with anti-phosphorylated pan-Akt (pAkt1/2/3) antibody and then reprobed with anti-pan-Akt antibody, anti-a-tubulin antibody. The figure shows data from one independent experiment (n = 3; separation bars indicate non-contiguous lanes on the same image acquisition (the original blots are shown in the Supplementary Information file).

The pattern of induction of downstream effector protein kinases (Erk1/2 and Akt) was examined to further characterize the activity profile of small BDNF mimetic GSB-106 in serum starved cells. As shown in Fig. 4A,B, Erk1/2 activation occurred at 180 min in the presence of GSB-106, whereas Erk1/2 phosphorylation, induced by BDNF, registered in 30 min, reaching a maximum increase at 180 min. Notably, a greater (1.5 fold; p < 0.05; Wilcoxon *t*-test) Erk1/2 activation at 180 min was produced by BDNF compared to GSB-106. Figures 4A,C demonstrate that treatment of serum free cells with BDNF led to robust raise in Akt phosphorylation at 30 min, followed by a decrease at 180 min (p < 0.05; Wilcoxon *t*-test). Similar, although less pronounced profile of Akt activation has been established upon GSB-106 exposure: a moderate stimulation of Akt phosphorylation at 30 min with a consequent kinase activation decline at a later time point.

Pretreatment of cells with a pharmacological Trk inhibitor K252a led to suppression of BDNF- or GSB-106-induced activation of Erk1/2 and Akt (Fig. 4D), thus suggesting that the pro-survival signaling evoked by GSB-106 in serum withdrawn SH-SY5Y cells requires TrkB activation. To further confirm the engagement of MAPK/Erk1/2 and PI3K/Akt pathways in GSB-106-mediated cell’s protection, cells were pretreated with PD98059 and LY294002. As shown in Fig. 4E PD98059 prevented both GSB-106 and BDNF-evoked activation of Erk1/2 phosphorylation and had no effect on the activity of Akt kinase (Supplementary Fig. S1A). Similarly, LY294002 abrogated the ability of GSB-106 and BDNF to stimulate the phosphorylation of Akt (Fig. 4F) and did not affect the phosphorylation of Erk1/2 (Supplementary Fig. S1B). Together, these findings indicate that GSB-106 promotes serum deprived cells survival through activation of TrkB and downstream MAPK/Erk1/2 and PI3K/Akt pathways.

### Activation of PI3K/Akt pathway is essential for GSB-106-mediated neuroprotection

**Figure 5:**
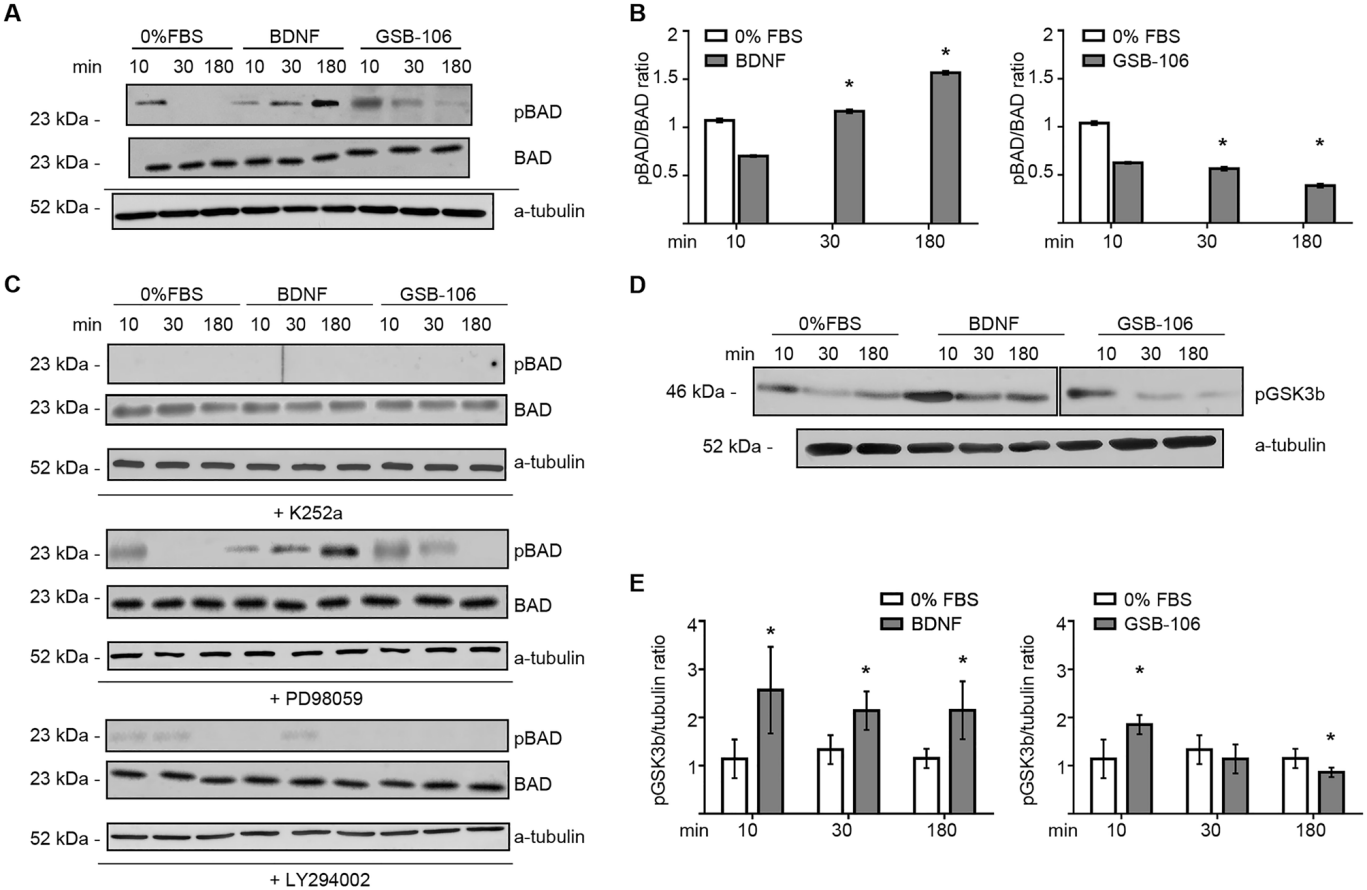
GSB-106-induced neuroprotection involves the BAD and GSK3β proteins. (**A**) GSB-106 activates Bad phosphorylation. SH-SY5Y cells were incubated in serum-free culture medium (“0% FBS”) with or without BDNF (~ 1 nmol) or GSB-106 (100 nmol) for 10 min, 30 min, or 180 min. Blots were first probed with anti-phosphorylated BAD (pBAD) antibody and then reprobed with anti-BAD antibody, anti-a-tubulin antibody. The figure shows data from one independent experiment (n = 3; the original blots are shown in the Supplementary Information file). (**B**) The ratio of signal derived from phosphorylated BAD over BAD bands was calculated (n = 3; *p < 0.05 in relation to “0% FBS”; Wilcoxon *t*-test). (**C**) SH-SY5Y cells in serum-free culture medium (“0% FBS”) with or without BDNF (~ 1 nmol) or GSB-106 (100 nmol) were also pre incubated with or without K252a (500 nmol, 60 min), LY294002 (50 µmol, 60 min) or PD98059 (50 µmol, 60 min). The figure shows data from one independent experiment (n = 2; the original blots are shown in the Supplementary Information file). (**D**) SH-SY5Y cells were incubated in serum-free culture medium (“0% FBS”) with or without BDNF (~ 1 nmol) or GSB-106 (100 nmol) for 10, 30, 180 min. Blots were first probed with anti-pGSK-3β antibody and then were reprobed with anti-a-tubulin antibody. The figure shows data from one independent experiment (n = 3; the original blots are shown in the Supplementary Information file). (**E**) The ratio of signal derived from p-GSK-3β over a-tubulin bands was calculated (n = 3; *p < 0.05 in relation to corresponding “0% FBS”; Wilcoxon *t*-test).

TrkB-dependent activation of the PI3K/Akt cascade regulates number of proteins essential for neuronal survival, growth and differentiation such as GSK-3β (glycogen synthase kinase 3β) and certain substrates that directly regulate the caspase cascade, in particular, the proapoptotic factor—BAD (Bcl-2 antagonist of cell death)^34^. The proapoptotic activity of BAD is regulated by several protein kinases (e.g. Akt, Rsk) through phosphorylation at sites known to inhibit its pro-apoptosis function and thus suppress BAD-mediated apoptosis in neurons^35^. We evaluated the effects of GSB-106 on activity of BAD, GSK-3β proteins and caspases-9, 3/7 in serum starved cells to characterize the survival-promoting and anti-apoptotic mechanisms of the BDNF mimetic in more detail. Figures 5A,B show that serum starvation results in a loss of phosphorylated BAD protein at 30 min, whereas incubation of cells with BDNF was accompanied with an incremental rise of BAD phosphorylation (Ser136) lasting for up to 180 min (p < 0.05; Wilcoxon *t*-test), which is consistent with conventional knowledge of BDNF ability to inhibit apoptosis through the BAD phosphorylation, along with other mechanisms^28^. GSB-106 has eliminated the serum withdrawal-induced decrease of BAD phosphorylation, maintaining the moderate level of pBAD within 180 min (p < 0.05; Wilcoxon *t*-test) as shown in Fig. 5A,B. Although the extent of BAD activation caused by GSB-106 was less prominent compared to BDNF, these findings still point out GSB-106 ability to mimic the neurotrophin-specific anti-apoptotic mechanism mediated by BAD protein.

Next, we determined the role of upstream components of TrkB/PI3K/Akt cascade in BAD phosphorylation, induced by BDNF and GSB-106. BDNF and GSB-106 both failed to phosphorylate BAD protein in the presence of K252a and LY294002 (Fig. 5C). Thus, TrkB and Akt are likely required for BDNF- and GSB-106-stimulated BAD activation in serum withdrawn SH-SY5Y cells. In contrast, BAD Ser136 phosphorylation was not altered by PD98059 treatment, corroborating the insensitivity of this site to activation by Ras/MAPK pathway (Fig. 5C). As it is known, MAPK-activated kinase Rsk (non-specific serine/threonine protein kinase) catalyzes the BAD phosphorylation at Ser112 followed by suppression of BAD-mediated apoptosis^36^.

We have evaluated phosphorylation of GSK-3β, a downstream target of PI3K/Akt aiming to learn about additional mechanisms of PI3K/Akt-dependent protection of SH-SY5Y cells from apoptotic cell death induced by serum deprivation under BDNF and GSB-106 treatment. Western blot analysis showed the decreased level of p-GSK-3β in serum-free SH-SY5Y cells which was reversed and remained elevated for 3 h upon BDNF treatment. Addition of GSB-106 to serum-deprived cells caused a transient increase of GSK-3β Ser9 phosphorylation peaking at 10 min followed by a gradual decline over the next 3 h (p < 0.05; Wilcoxon *t*-test; Fig. 5D,E).

**Figure 6:**
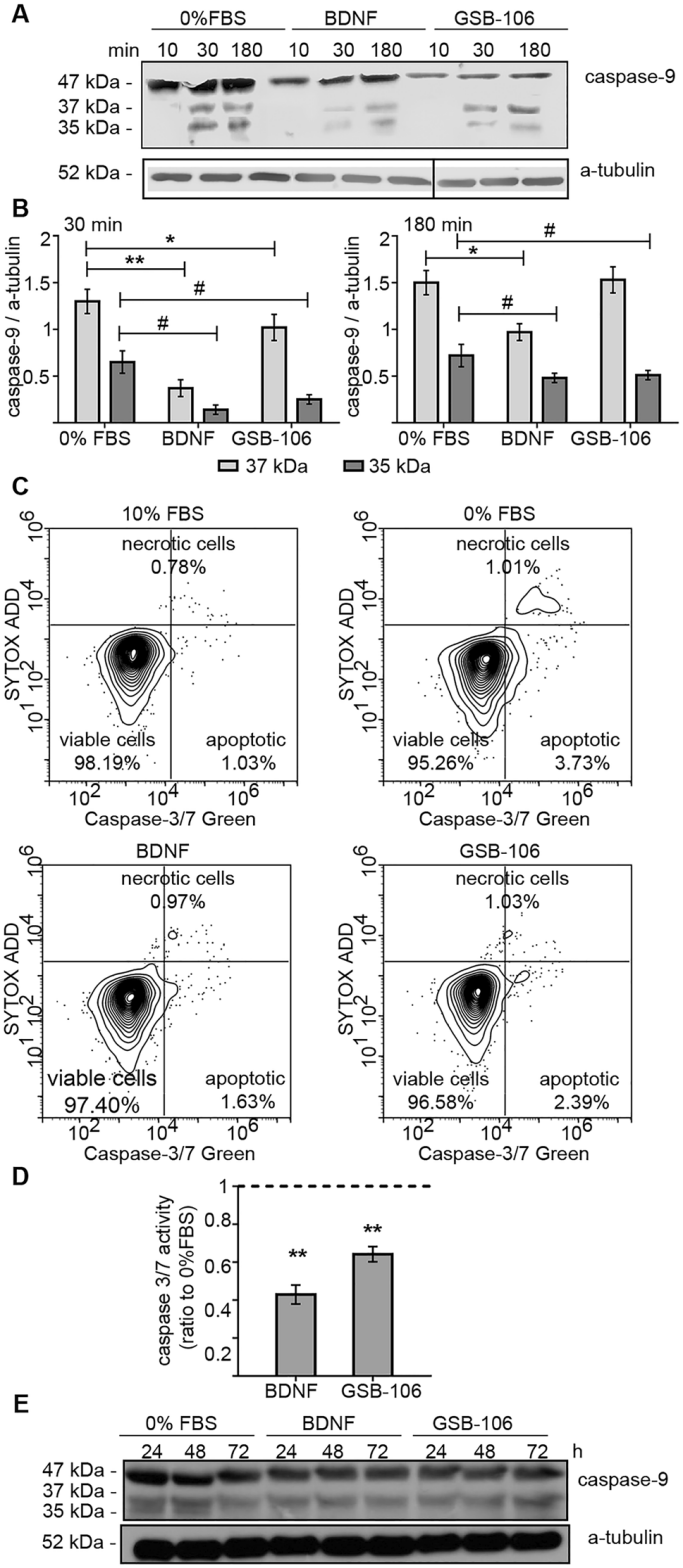
GSB-106 inhibits caspase-9 and caspase-3/7 activities induced by in serum deprivation. (**A**) SH-SY5Y cells were incubated in serum-free culture medium (“0% FBS”) with or without BDNF (28.6 ng/ml, ~ 1 nmol) or GSB-106 (100 nmol) for 10, 30, 180 min. After incubation, cells were collected and protein extracts were subjected to polyacrylamide gel electrophoresis and transferred for Western blotting. Blots were first probed with anti-caspase-9 antibody and then reprobed with anti-a-tubulin antibody. The figure shows data from one independent experiment (n = 3; the original blots are shown in the Supplementary Information file). (**B**) The ratio of signal derived from caspase-9 over a-tubulin bands was calculated (n = 3; *p < 0.05, **p < 0.01 in relation to 37 kDa caspase-9 form in “0% FBS” group; ^#^p < 0.05 in relation to 35 kDa caspase-9 form in “0% FBS” group; Wilcoxon *t*-test). (**C**) SH-SY5Y cells were incubated in serum-free culture medium (“0% FBS”) with or without BDNF (28.6 ng/ml, ~ 1 nmol) or GSB-106 (100 nmol) for 6 h. After incubation, cells were collected and stained with or CellEvent Caspase-3/7 Green Flow Cytometry Assay Kit (C10427; Thermo Fisher Scientific) according manufacture’s recommendations. The figure shows contour plots from one independent experiment (n = 3). (**D**) The ratio of CellEvent Caspase-3/7 Green fluorescence signals derived from caspase3/7^+^/SYTOX^−^ cell populations was calculated. Data is expressed as means ± S.E.M. Three independent experiments were carried out in triplicate (**p < 0.01 vs “0% FBS” (dashed line); Wilcoxon *t*-test). (**E**) GSB-106 induced a long-term decrease of caspase-9 activity. SH-SY5Y cells were incubated in serum-free culture medium (“0% FBS”) with or without BDNF (28.6 ng/ml, ~ 1 nmol) or GSB-106 (100 nmol) for 24, 48, 72 h. After incubation, cells were collected and protein extracts were subjected to polyacrylamide gel electrophoresis and transferred for Western blotting. Blots were first probed with anti-caspase-9 antibody and then reprobed with anti-a-tubulin antibody. The figure shows data from one independent experiment (n = 3; the original blots are shown in the Supplementary Information file).

It is well known that intrinsic apoptotic pathway triggered in response to withdrawal of growth factors, results in the mitochondrial release of cytochrome *c*, which activates initiator caspase-9. Once activated, caspase-9 cleaves and induces executioner caspase-3^37^. Figures 6A,B show that serum deprivation increased cleaved caspase-9 (detected by appearance of processed caspase-9 which yielded p35 and p37 subunits) within short time period, beginning at 30 min and maintaining for up to 180 min, and for a prolonged period lasting up to 72 h, thus implying permanent activation of caspase-9 (Fig. 6E). The decrease in p35 and p37 of caspase-9 subunits levels has been observed in the presence of both BDNF and GSB-106 at an early time point (p < 0.05, p < 0.01; Wilcoxon *t*-test; Fig. 6A,B). This reduction continued for 72 h, reflecting the sustained supression of caspase-9 activity (Fig. 6E). It is remarkable that time course of caspase-9 inhibition is consistent with the dynamic of Akt and BAD activation elicited by both GSB-106 and BDNF, which suggests that GSB-106 promotes suppression of apoptosis exerted by serum withdrawal through Akt-dependent protection mechanisms, which are attributive to BDNF. Moreover, these data further corroborated the neurotrophin-like mode of neuroprotection, displayed by GSB-106.

Caspase-3/7 activity was directly assessed by CellEvent Caspase-3/7 Green Flow Cytometry Assay Kit in order to further evaluate the effect of GSB-106 and BDNF on downstream apoptotic pathway. This detection reagent consists of a nucleic acid-binding dye that harbors the caspase-3/7 cleavage sequence DEVD and is fluorescent after being cleaved and bound to DNA. As observed in Fig. 6C,D, activated (cleaved) caspases-3/7 signals decreased after BDNF and GSB-106 treatment by 57.0 ± 5% and 35.8 ± 4% respectively in comparison with signals in control serum deprived cells (p < 0.01; Wilcoxon *t*-test). These findings indicate that GSB-106 and BDNF attenuate activity of caspases-9 and 3/7, thus supporting antiapoptotic signaling.

## Discussion

This study reports on BDNF small dipeptide mimetic GSB-106 being able to protect trophic-deprived cells from apoptosis and promote their survival by triggering the TrkB-dependent activation of downstream pro-survival pathways. Current study also suggests that GSB-106 behaves as a partial BDNF-like agonist, since GSB-106 was found to intrinsically provide the cell survival (~ 26% increase over control) and is able to inhibit BDNF-mediated cell viability (by ~ 37%), when added competitively, thus exhibiting the profile of partial agonist. Remarkably, an increase in GSB-106-promoted survival effect in the presence of BDNF, compared to cells, treated by GSB-106 alone, has been observed, suggesting the additional (or parallel) mechanisms of cell viability promotion upon serum withdrawal conditions.

Studied compound reproduces some functions inherent for BDNF. Particularly, neuroprotection of serum-deprived cells, provided by GSB-106, involved inhibition of apoptosis and was shown to be dependent on a transient TrkB receptor activation, which was assayed by phosphorylation at Tyr^706/707^. Furthermore, GSB-106 increased TrkB phosphorylation at Tyr^515^ and Tyr^816^ and promoted activation of Ras/MAPK and PI3K/Akt pathways. Patterns for Erk1/2 and Akt activation induced by GSB-106 were weaker, than those triggered by BDNF. However, it had similar temporal pattern, and this activation was prevented by K252a kinase inhibitor and corresponding MEK1 and PI3 kinase inhibitors (PD98059 and LY294002 respectively). Intriguingly, Tyr^515^ phosphorylation was affected by GSB-106 more significantly, than that induced by BDNF; particularly, the enhanced level of Tyr^515^ activation has already appeared at 10 min (~ 64%) and maintained up to 60 min, although BDNF elicited an increase of phosphorylation at this site (~ 38%) only after 60 min. While TrkB Tyr^706/707^ and Tyr^816^ phosphorylation were almost completely eliminated by K252a, a residual Tyr^515^ phosphorylation has retained upon both BDNF and GSB-106 treatment, hence, suggesting the contribution of non-TrkB-dependent mechanisms. Experiments with Src kinases inhibitor PP2 showed that Src kinases, apparently, implicated in GSB-106-induced TrkB activation and, potentially, transactivation mechanisms could account for elevated pro-survival response seen during cell treatment by GSB-106 in the presence of BDNF.

Most importantly, protection of serum-deprived cells by GSB-106 also engages TrkB/Akt-dependent inactivation of pro-apoptotic BAD protein and suppression of caspases-9 and 3/7. Once activated by trophic factors, e.g. NGF (nerve growth factor) and BDNF, Akt phosphorylates BAD at Ser136, which in turn, sequestered in the cytosol in the complex with chaperone 14-3-4 protein and allows anti-apoptotic Bcl-2/Bcl-x_L_ proteins inhibit the activity of pro-apoptotic Bax protein, thereby preventing release of mitochondrial cytochrome *c*, activation of caspase cascade, thus promoting cell survival^34^. Moreover, GSB-106 increases the phosphorylation of GSK-3β at Ser9 transiently, to a substantially lesser extent than BDNF, thus inactivating the kinase; this mechanism shown to be involved in protective function of PI3K/Akt pathway^38^. Collectively, it appears that survival-promoting effect of GSB-106 as well as of BDNF in serum-deprived cell is related to TrkB-dependent activation of MAPK/Erk1/2 and, mainly, PI3K/Akt pathways. Furthermore, PI3K/Akt—dependent inactivation of pro-apoptotic BAD protein followed by subsequent downregulation of appropriate caspases could account for the protective mechanisms governed by the compound. Presumably, inactivation of GSK-3β is also relevant to protective mechanisms provided by GSB-106. There is no current evidence that existing small peptide or non-peptide BDNF mimetics promote survival through inactivation of BAD proteins in various damaging cell context considering the available data. Hereby, this is the first time when BDNF peptide mimetic effect has been identified to require the TrkB/Akt-dependent inactivation of BAD protein in serum-deprived cells to support the survival.

GSB-106 produced a bell-shaped concentration–response survival curve in serum-deprived SH-SY5Y cells, which is generally a characteristic of compounds that mimic receptors agonist ligands, capable of homodimerization, mainly of receptors of growth factors, some hormones and cytokines^39,40^. Bell-shaped concentration–response curves and partial agonist profiles have been also reported for a number of domain-specific small molecule mimetics of BDNF, which are capable of engaging with and induce the dimerization and/or activation of TrkB receptor^13,41–43^. Several mechanistic models have been rendered to explain how the small neurotrophin peptidomimetics and non-peptide ligands can elicit the TrkB-dependent cellular response, although the exact structural basis for peptidomimetics/Trks interaction and subsequent activation remain obscure^44^. Under conventional interpretation, small monomeric or dimeric molecules activate Trks upon binding by inducing receptor dimerization. There is also a possibility that small neurotrophin-like ligands might interact with existing pre-formed, yet inactive Trk dimers or Trk/p75^NTR^ heterodimers. It was suggested that neurotrophin mimetics could stabilize preformed dimers, hence, encourage the receptor activation, considering such a mode of natural ligands action. Furthermore, additional option is associated with probable allosteric regulation of Trk receptors conferred by low molecular mass neurotrophin mimetics^44^.

Taking into account structural features of homodimeric dipeptide GSB-106, such as resemblance of one of the TrkB-interacting motif (amino acids 4 loop of BDNF -Asp^93^-Ser^94^-Lys^95^-Lys^96^-), presence of dimerizing linkage (oligomethylenediamine spacer) and functional in vitro and in vivo data, it is feasible that GSB-106 capable of binding to functional sites of TrkB and promoting or facilitating dimerization of the receptor, which provides the conformational changes, sufficient to trigger at a certain extent receptor autophosphorylation followed by activation of downstream signaling pathways. Interestingly, **-Ser-Lys-** motif occurred in both sites of neurotrophin (within the 2 loop: -**Ser**^**45**^**-Lys**^**46**^-Gly^47^-Gln^48^-Lys^49^- and within the 4 loop: -Asp^93^-**Ser**^**94**^**-Lys**^**95**^-Lys^96^-), which are critical for BDNF/TrkB binding and activity and possess functional importance. As noted above, this motif represents core dipeptide fragment of GSB-106 that, probably, affords BDNF-like trophic activity of the compound. Conceivably, bell-shaped concentration–response curve could reflect the ability of GSB-106 to bind and stimulate/support receptor dimerization. Nevertheless, given that GSB-106 was designed to mimic only one of multiple BDNF/TrkB-interacting domains, incomplete receptor binding could arise, thus contributing to conformational changes distinct from those inherent for BDNF. That may result in a fewer degree of receptor and effector kinases activations and temporal discrepancy of receptor phosphorylation patterns, compared to that of BDNF. Presumably, partial agonist profile of the GSB-106 could represent different from BDNF temporal and activation patterns of TrkB phosphorylation, reduced activation of downstream effectors and less pronounced inactivation of BAD and GSK-3β. However, mechanisms, underlying the partial agonistic effect and the bell-shaped curve evoked by GSB-106 require further investigations.

Considering that residues located in the loop 4 BDNF (Lys^95^, Lys^96^, Arg^97^) appeared to contribute both to TrkB and p75^NTR^ binding^45^, we do not rule out the possible interaction of GSB-106 with p75^NTR^, although it is unlikely that compound could interact with both receptors simultaneously^46,47^, whilst this issue has not been addressed in the current study. Insofar we did not evaluate the direct interaction of GSB-106 with TrkB, as well as with p75^NTR^, the role that distinct receptor plays in pro-survival properties of GSB-106 couldn't be precisely resolved and requires further detailed investigations. Nevertheless, several lines of indirect evidence allow us to speculate that GSB-106 could function preferentially through the TrkB -, rather than, p75^NTR^—dependent pathways. First, serum-starved SH-SY5Y cells co-express TrkB and p75^NTR^ receptors (Fig. 1B), therefore, TrkB-dependent survival signaling would be prevalent following neurotrophin addition^3^. Indeed, GSB-106 alone acts in a manner similar to that of BDNF in a such cellular context, rendering cell survival, which is partially TrkB-dependent and accompanied by TrkB Tyr^707/707^, Tyr^516^, Tyr^817^ phosphorylation and TrkB-regulated activation of MAPK/Erk and PI3K/Akt signaling. Second, in instances when TrkB and p75^NTR^ expressed concomitantly, Trks suppress the p75^NTR^-mediated apoptotic JNK-p53-Bax pathway through Ras/PI3K/Akt and Ras/MAPK/Erk pathways or inhibit cell death proteins activities, such as Forkhead and members of the Bcl-2 family (Bcl-2, Bcl-xL, Bim and BAD)^6^. As it was mentioned above, growth factors-promoted inhibition of apoptosis, mediated by Akt activation, results in phosphorylation of BAD at Ser 112, 136 and 155, thus decreasing BAD association with Bcl-xL followed by inhibition of pro-apoptotic Bax protein and caspases-9, 3/7, thereby promoting cell survival^34^. Our data showed that GSB-106, as well as BDNF, prevented apoptosis, induced by serum-withdrawal, mainly via TrkB/Akt-mediated phosphorylation (Figs. 4D, 5C) and inactivation of the proapoptotic BAD protein (Fig. 5A) with subsequent suppression of downstream initiator (caspase-9) and effector (caspases-3/7) caspases (Fig. 6). Obtained results suggest, that pro-survival activity of GSB-106 could also relay on the counteraction of deleterious pathways arising from p75^NTR^, although, this assumption requires faithful delineation.

Based on our findings we also presumed indirect TrkB activation by GSB-106 in serum-deprived SH-SY5Y cells, which could explain the gain (by ~ 17%) of GSB-106 survival activity in the presence of BDNF. TrkB signaling undergoes transactivation, which involves several GPCRs (G-protein-coupled receptors), such as A_2A_ adenosine receptors^32^, PACAP (pituitary adenylate cyclase-activating polypeptide) receptors PACR1^48^, dopamine D_1_ receptors^49^, cannabinoid receptors CB_1_R^50^. BDNF-independent TrkB-activation also occurs via epidermal growth factor (EGF)^51^, glucocorticoids^52^, zinc^53^, H_2_O_2_^54^. The finding by Lee and Chao provided evidences of Src kinases implication in phosphorylation of intracellular TrkB tyrosines and receptor activation^32^. Huang and McNamara carefully elucidated the role of SFKs in activation of TrkB by zinc and BDNF. It has been reported that BDNF activation of TrkB resulted in increased SFKs activity, promotion of protein complex formation consisting of TrkB and SFKs—Fyn and Src in vitro. Additional experimental data supported the hypothesis that SFKs can provide the positive feedback regulation of TrkB signaling provoked by BDNF^33^. According to our data, SFKs inhibitor PP2 suppressed the GSB-106-evoked Tyr^706/707^, Tyr^515^ and Tyr^816^ phosphorylation, thus supporting the assumption that recruitment of SFKs required for GSB-106-regulated TrkB activation, yet not excluding other non-TrkB targets or mechanisms which could be responsible for improving of cell survival, stimulated by GSB-106 in the presence of BDNF. Remarkably, SFKs inhibition led to a smaller reduction of BDNF-induced Tyr^706/707^ and Tyr^515^ phosphorylation than that produced by GSB-106, although decrease in Tyr^816^ phosphorylation was more profound. Distinct patterns of different TrkB tyrosines phosphorylation inhibition imply that BDNF and GSB-106 could function non-identically on certain sites via involvement of divergent mechanisms. To insight into mechanisms of GSB-106 action further analysis of GSB-106/TrkB and GSB-106/p75^NTR^ interactions, possible non-TrkB targets and associated signaling pathways will be needed.

In summary, this report shows that GSB-106, similarly to BDNF, promotes survival of serum-deprived neuronal-like cells. This protective effect is governed by the ability of compound to counteract cell apoptosis through activation of TrkB-dependent pro-survival mechanisms, including inactivation of pro-apoptotic BAD protein and suppression of caspases-9 and 3/7. We demonstrated that GSB-106 acted as a partial TrkB receptor agonist and transactivation mechanisms also could contribute to pro-survival properties of the compound. Taken together, multiple lines of evidence indicate that GSB-106 functions as BDNF-like TrkB ligand mimicked the pro-survival activity and the principal protective mechanisms inherent for the native neurotrophin.

## Materials and methods

### Substance

GSB-106 (bis-(*N*-monosuccinyl-l-seryl-l-lysine) hexamethylenediamide; MW 746.85) was synthesized as described previously [Fig. 1A; 15] at Medicinal Chemistry Department of “Zakusov Institute of Pharmacology”. GSB-106 and BDNF were dissolved in Dulbecco’s modified Eagle’s medium (DMEM) without serum for all experiments.

### Cells and reagents

Neuroblastoma cell line SH-SY5Y was kindly provided by Dr. Galina Pavlova (Institute of Gene Biology Russian Academy of Sciences, Moscow). Cells (at passages 2–10) were maintained in DMEM, supplemented with 10% fetal bovine serum (FBS), 10^–3^ M l-glutamine in a 5% CO_2_ incubator at 37 °C. In cell-based assays, SH-SY5Y cells were seeded on 48-, 12- or 6-well plates (Corning). After reaching 85–95% confluency, cells were gently washed with PBS (2–3 times) and starved in 0% FBS DMEM for at least 24 h (unless otherwise specified). Cells were incubated with BDNF or GSB-106 (in one or various concentrations, depending on the type of experiment) for different time intervals after starvation. Chemicals and cell culture reagents were obtained from Sigma-Aldrich, Thermo Fisher Scientific, Tocris.

### Antibodies

Following primary antibodies were used in this study: rabbit polyclonal anti-BDNF (1:1000, #ab226843, Abcam), rabbit monoclonal anti-TrkB (1:1000, #4603, Cell Signaling Technology), anti-p75^NTR^ (1:1000, #4201, Cell Signaling Technology), anti-phospho-TrkA (Tyr674/675)/TrkB (Tyr706/707) (1:1000, #4621, Cell Signaling Technology), anti-p44/42 MAPK (Erk1/2) (1:1000, #4695, Cell Signaling Technology), anti-phospho-p44/42 MAPK (Erk1/2) (Thr202/Tyr204) (1:1000, #4377, Cell Signaling Technology), anti-Akt (pan) (1:1000, #4691, Cell Signaling Technology), anti-phospho-Akt (Ser473) (1:1000, #4058 Cell Signaling Technology), anti-BAD (1:1000, #9239, Cell Signaling Technology), anti-phospho-BAD (Ser136) (1:1000, #4366, Cell Signaling Technology), anti-phospho-GSK-3β (Ser9) (1:1000, #9323, Cell Signaling Technology), rabbit polyclonal anti-phospho-TrkB (Tyr816) (1:1000, #NBP1-03499SS, Novus Biologicals), anti-phospho-TrkB (Tyr515) (1:1000, # PA5-36695, Thermo Fisher Scientific), anti-Caspase-9 Antibody (1:1000, #9502, Cell Signaling Technology), mouse monoclonal anti-α-Tubulin (1:1000, #2125, Cell Signaling Technology). Secondary antibodies conjugated to HRP (used at 1:12,000 dilution) were from Cell Signaling Technology (anti-mouse IgG, #7076; anti-rabbit IgG, #7074).

### MTT assay (viability assay)

SH-SY5Y cells were seeded in 48-well plates (Corning) (2 × 10^5^ cells/200 µl/well) in DMEM containing 10% FBS, 10^–3^ M l-glutamine in a 5% CO_2_ incubator at 37 °C. Cells were gently washed with PBS (2–3 times) upon reaching 85–95% confluency. Media was changed to DMEM (0% FBS), containing exogenous BDNF (1–2–4–10–100 nmol; #B3795, Sigma-Aldrich) or/and GSB-106 (0.1 nmol to 100 µmol) after washing. For TrkB—and downstream signaling pathways—mediated survival assays cells were incubated with BDNF (1 nmol) or GSB-106 (100 nmol) in the presence either of K252a (500 nmol), or PP2 (100 μmol), or PD98059 (50 μmol), or LY294002 (1 μmol). Cells were cultured for 48 h. 20 μl of the MTT reagent (3-(4,5-dimethylthiazol-2-yl)-2,5-diphenyl tetrazolium bromide; final concentration 0.5 mg/ml; #M6494, Thermo Fisher Scientific) were added to each well after incubation period was completed. Cells were incubated for 4 h in a humidified atmosphere. Cultural media was completely removed, and the pellets were solubilized in 100% DMSO (Sigma-Aldrich) for 10 min in room temperature. Absorbance was assessed using Varioskan LUX microplate reader (Thermo Fisher Scientific) after purple formazan crystals fully dissolved. Absorbance of the formazan product was measured at 590 nm wavelength. 750 nm reference wavelength was used. 590/750 nm ratio of absorbance was used (A_590/750_) for further analysis. After BDNF or GSB-106 treatment cell viability rates were calculated as follows:

$${\text{Cell}}\,{\text{viability}},\% = \frac{{({\text{A}}590/750^{{{\text{treatment}}}} - {\text{A}}590/750^{{{\text{serum}}\,{\text{free}}}} )}}{{{\text{A}}590/750^{{{\text{serum}}\,{\text{free}}}} }} \times 100$$

The comparative analysis of cell survival upon BDNF, GSB-106 and BDNF + GSB-106 incubation, as well as analysis of GSB-106-dependent viability upon K252a, PP2, PD98059, LY294002 treatments was performed as follows:

$$\% \,{\text{BDNF}}\,{\text{maximum}}\,{\text{survival}} = \frac{{({\text{A}}590/750^{{{\text{treatment}}}} - {\text{A}}590/750^{{{\text{serum}}\,{\text{free}}}} )}}{{({\text{A}}590/750^{{{\text{BDNF}}}} - {\text{A}}590/750^{{{\text{serum}}\,{\text{free}}}} )}}$$

### Protein preparation and Western blot analysis

SH-SY5Y cells were cultured in 6-well plates for 24 h (Corning) (3 × 10^6^ cells/well) in DMEM, containing 10% FBS and after washing cells were maintained in serum-free DMEM. Neurotrophin or compound was added at different time points, depending on the experiment. At the definite time points, cells were lysed with RIPA buffer (10^–2^ M Tris–Cl (pH 8.0), 10^–3^ M EDTA, 5 × 10^–4^ M EGTA, 1% Triton X-100, 0.1% sodium deoxycholate, 0.1% SDS, 0.14 × 10^–3^ M NaCl, 1 × protease inhibitor cocktail (#P8340), 1 × phosphatase inhibitor cocktail 2 and 3 (#P5726, P0044), 10^–3^ M PMSF (all obtained from Sigma-Aldrich). Protein concentration was determined using BCA Protein Assay Kit (#23225, Thermo Fisher Scientific). Lysates were loaded on SDS-PAGE, and separated proteins were transferred onto the nitrocellulose membranes (#GE10600002, Sigma-Aldrich). The membranes were blocked in 5% not-fat milk or 5% BSA in TBS-T for 60 min at room temperature, incubated with the appropriate primary antibody (+ 4 °C, overnight) and then with a secondary antibody conjugated with HRP (60 min at room temperature). Bands were visualized using SignalFire Plus ECL Reagent (#12630, Cell Signaling Technology). Membranes were scanned using Amersham Imager 680 (GE Healthcare) and quantified in Image Quant TL v.8.1 (GE Healthcare). Measurement of each protein was then normalized on the related α-tubulin loading control.

### Caspase 3/7 activity assay

SH-SY5Y were cultured in 12-well plates (Corning) (5 × 10^5^ cells/well) in DMEM, containing 10% FBS for 24 h for flow cytometry detection of activated caspase-3 and caspase-7 in apoptotic cells. Cells were washed in PBS and maintained in serum-free DMEM for various time intervals upon BDNF or GSB-106 treatment. After incubation cells were harvested and stained with CellEvent Caspase-3/7 Green Flow Cytometry Assay Kit (#C10427, Thermo Fisher Scientific) according to the manufacturer’s recommendations. The samples were analyzed by NovoCyte 2060 flow cytometer (Agilent Technologies), using 488 nm excitation and collecting fluorescence emission; a 530/30 bandpass filter for CellEvent Caspase-3/7 Green Detection Reagent and a 690/50 BP filter for SYTOX AADvanced dead cell staining. After a standard fluorescence compensation technique, percent of caspase-3/7^+^/ SYTOX^−^cell populations on a dual parameter dot plot was used for statistical analysis (30,000 events were collected in each probes gated as “live cells”).

### Statistical analysis

Western blots data were statistically analyzed using Wilcoxon *t*-test (Statistica 12.5 (StatSoft. Inc.). Data is expressed as means ± S.E.M. Graphs plotting and IC_50_, EC_50_, EFFECTMAX calculations were carried out in the “bell-shape module” (nonlinear regression) of GraphPad Prism v.7.0 (GraphPad Software Inc.) for cell viability assays data. Comparison of survival curves was analyzed by one-way ANOVA with Newman-Keul’s post-hoc. Normal distribution of data was evaluated by the Shapiro–Wilk’s test. Levene’s test was used to check equality of dispersion.

## Supplementary Information

Supplementary Information.
