# Supplementary material for: Dimeric mimetic of BDNF loop 4 promotes survival of serum-deprived cell through TrkB-dependent apoptosis suppression
Source: Sci Rep. 2021 Apr 8;11:7781. doi: 10.1038/s41598-021-87435-0 (PMC8032782; doi:10.1038/s41598-021-87435-0)

Dimeric mimetic of BDNF loop 4 promotes survival of serum-deprived cell through TrkB-dependent apoptosis suppression

L.F. Zainullina [§], Yu.V. Vakhitova [§]*, A.Yu. Lusta, T.A. Gudasheva, S.B. Seredenin

Federal State Budgetary Institution «Research Zakusov Institute of Pharmacology»,

Moscow, Russia

[§]authors contributed equally to this work.

***Correspondence:** Vakhitova Yulia, vakhitova@academpharm.ru; Department of Pharmacogenetics, «Research Zakusov Institute of Pharmacology», Baltiyskaya str., 8, Moscow, 125315, Russia

Supplementary Info File

Data availability statement: All the original data acquisition of western blots is included in Supplementary Info file.

Keywords: BDNF, low weight mimetics, TrkB signaling, neuroprotection, BAD, GSK-3β

Supplementary Figure Legend

Supplementary Figure 1. (**A**) MEK1 inhibitor (PD98059) had no effect on Akt pathway. SH-SY5Y cells in serum-free culture medium («0% FBS») with or without BDNF (~ 1 nmol) or GSB-106 (100 nmol) were also pre incubated with or without PD98059 (50 µmol, 60 min). Blots were first probed with anti-phosphorylated pan-Akt (pAkt1/2/3) antibody and then reprobed with anti-pan-Akt antibody, anti-a-tubulin antibody. The figure shows data from one independent experiment (n=3; the original blots are shown in the supplementary information file).

(**B**) PI3K inhibitor (LY294002) had no effect on MAPK pathway. SH-SY5Y cells in serum-free culture medium («0% FBS») with or without BDNF (~ 1 nmol) or GSB-106 (100 nmol) were also pre incubated with or without LY290042 (50 µmol, 60 min). Blots were first probed with anti-phosphorylated Erk (pErk) antibody and then reprobed with anti-Erk antibody, anti-a-tubulin antibody. The figure shows data from one independent experiment (n=3; the original blots are shown in the supplementary information file).

Zainullina et al., Supplementary Figure 1

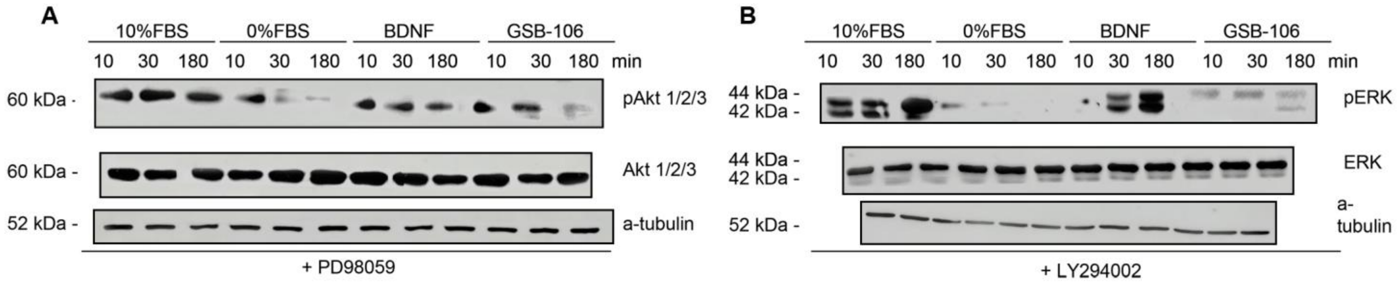

Figures with relative original data acquisition

Zainullina et al., Figure 1 as submitted

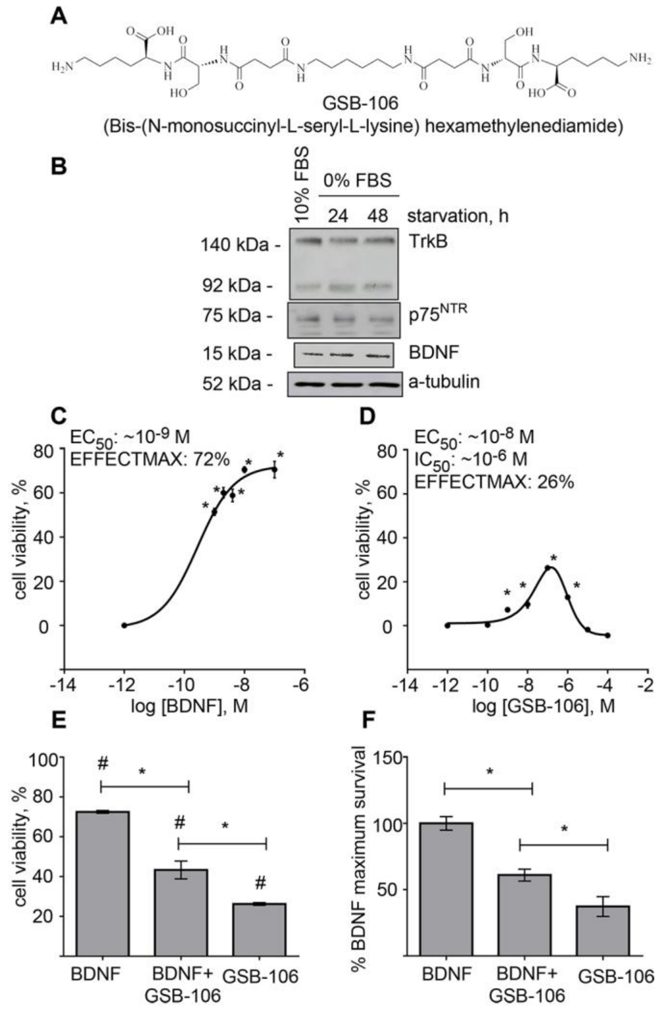

Zainullina et al., Figure 1

Original scans of blots, corresponding to figure 1B

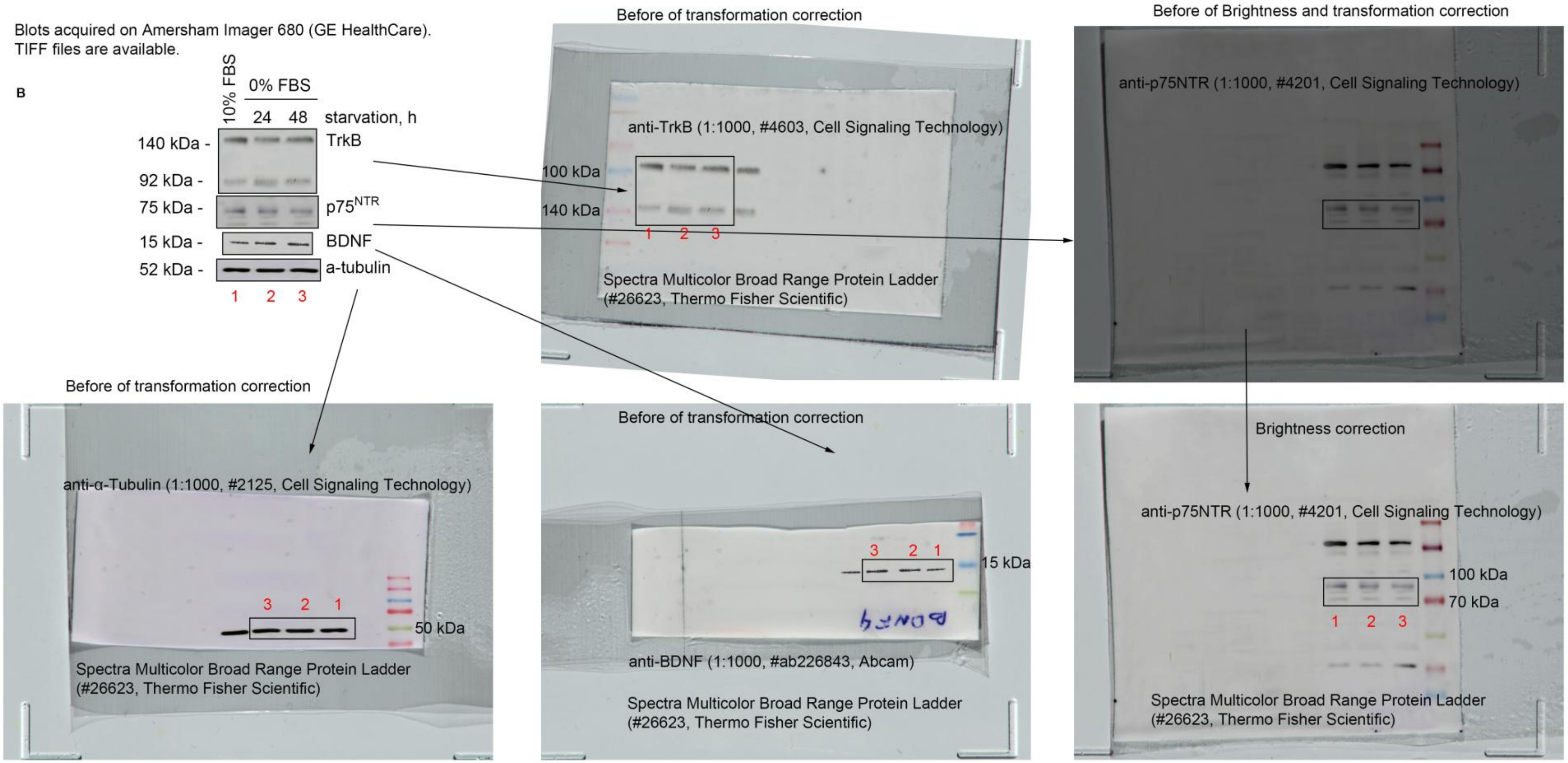

Zainullina et al., Figure 3 as submitted

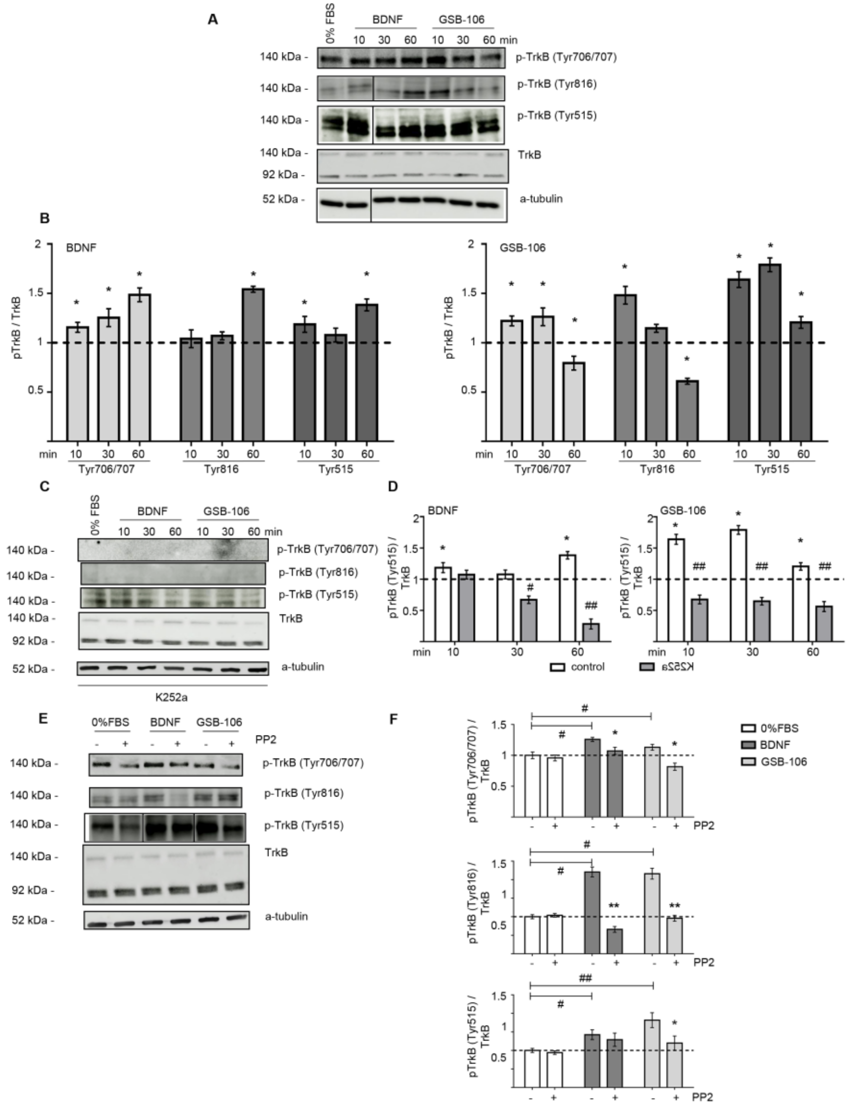

Zainullina et al., Figure 3

Original scans of blots, corresponding to figure 3A

Blots acquired on Amersham Imager 680 (GE HealthCare).
TIFF files are available.

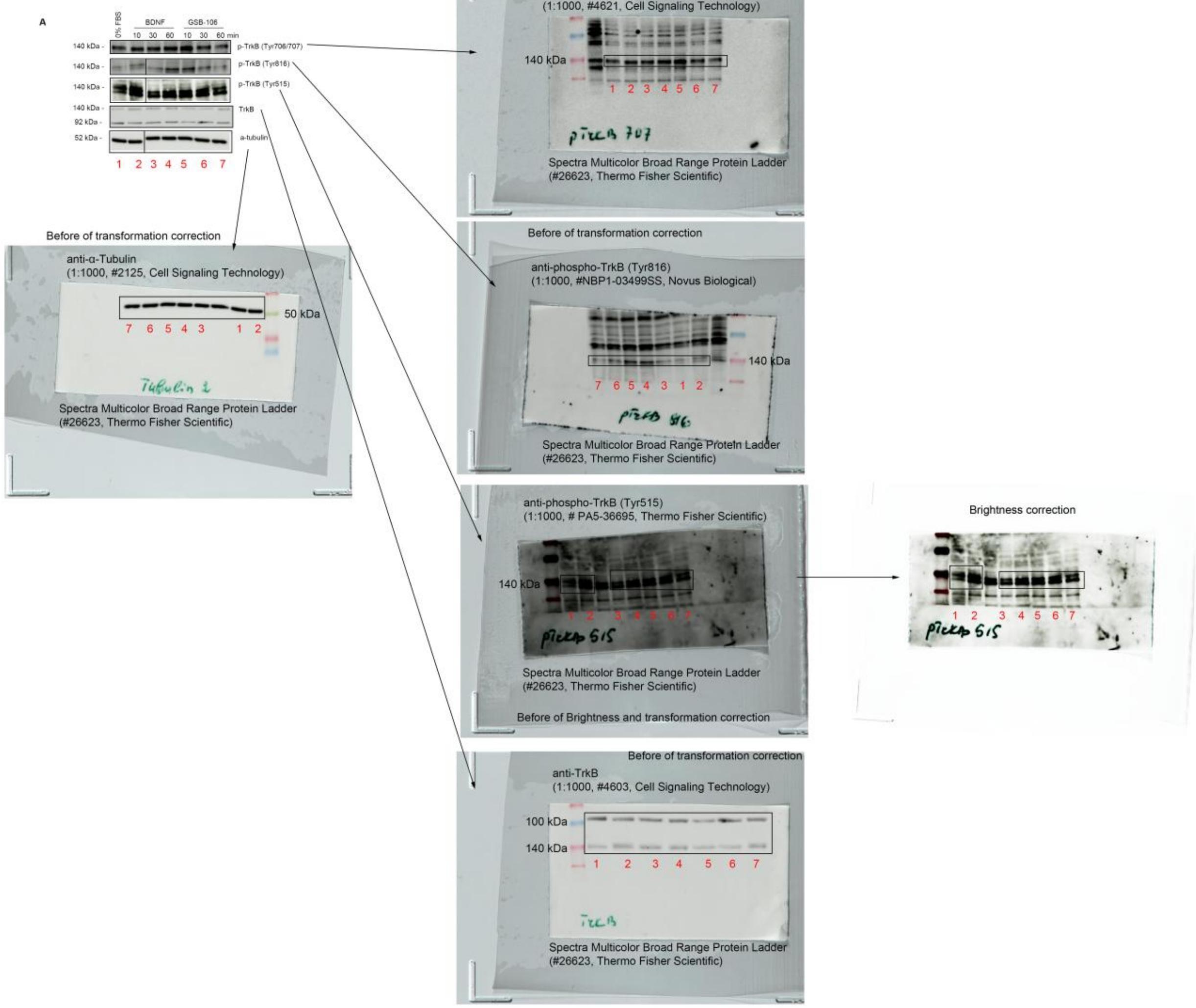

Zainullina et al., Figure 3

Original scans of blots, corresponding to figure 3C

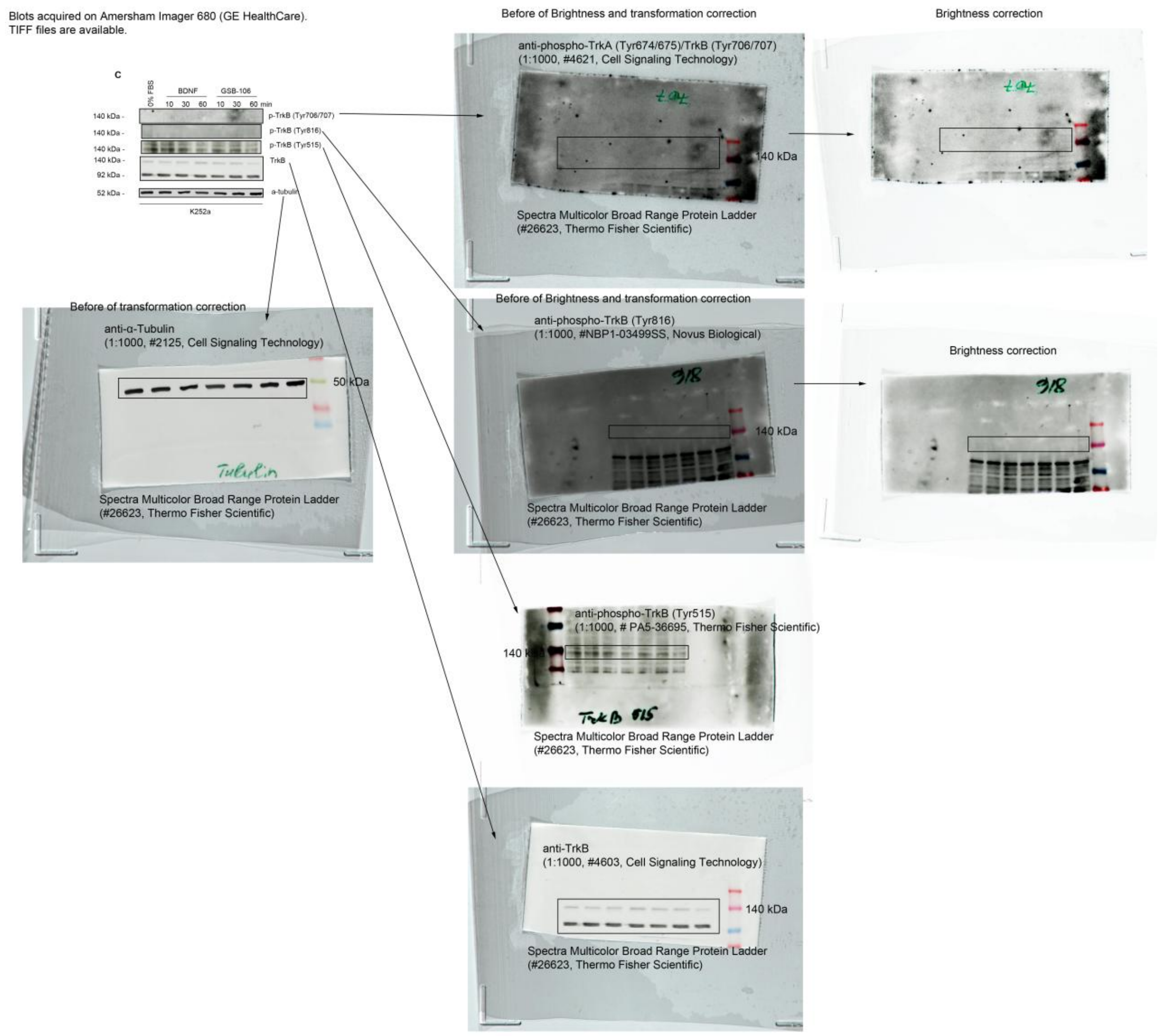

Zainullina et al., Figure 3

Original scans of blots, corresponding to figure 3E

Blots acquired on Amersham Imager 680 (GE HealthCare).
TIFF files are available.

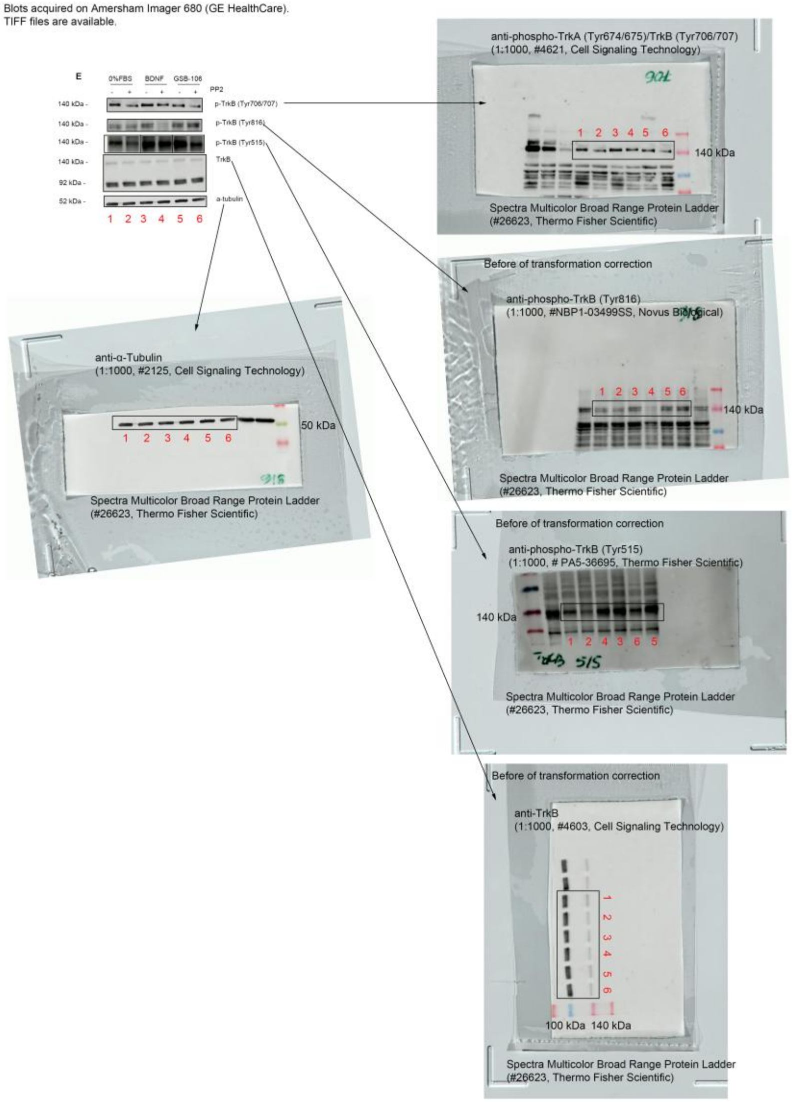

Zainullina et al., Figure 4 as submitted

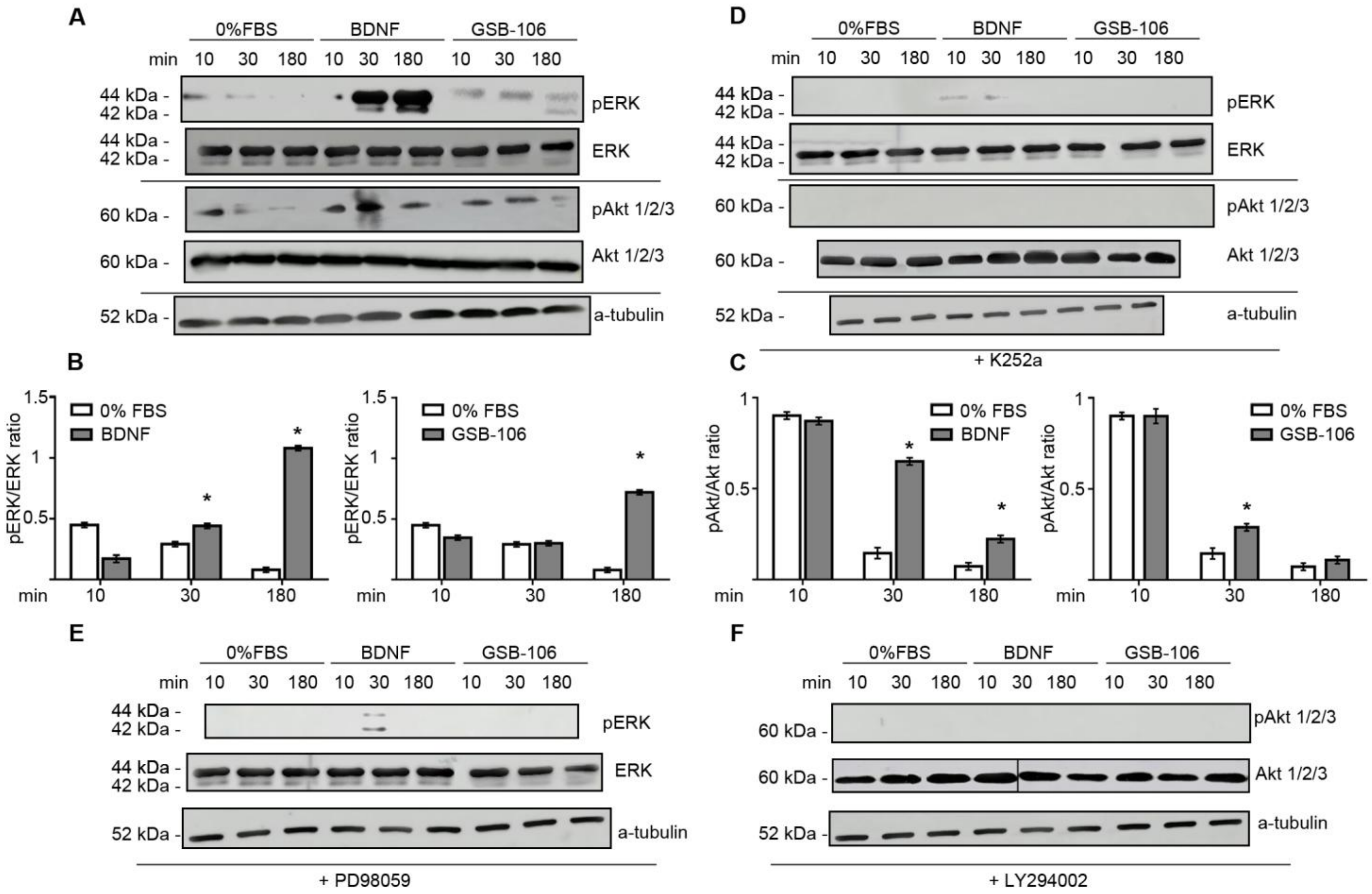

Zainullina et al., Figure 4

Original scans of blots, corresponding to figure 4A

Blots acquired on Amersham Imager 680 (GE HealthCare).
TIFF files are available.

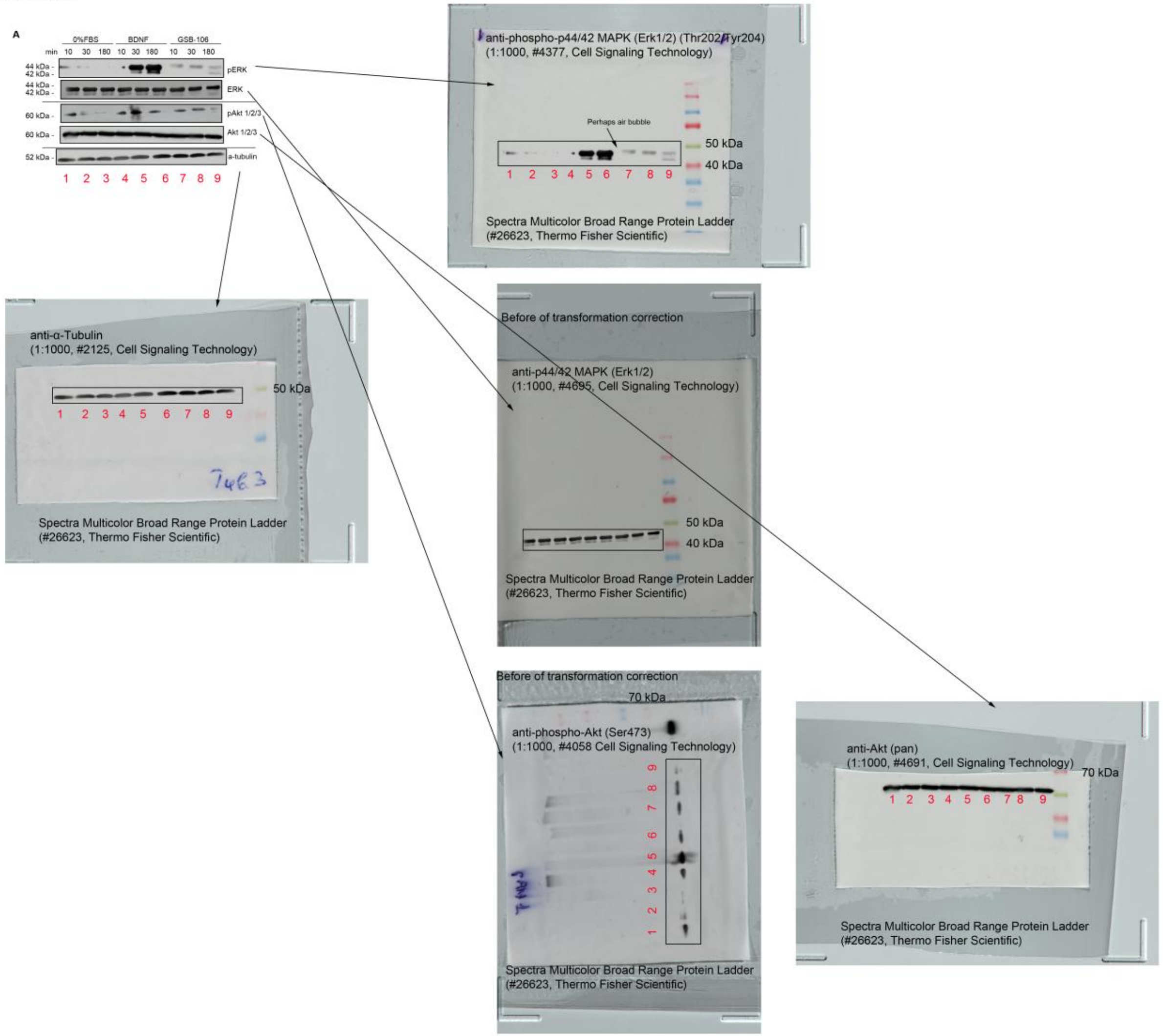

Zainullina et al., Figure 4

Original scans of blots, corresponding to figure 4D

Blots acquired on Amersham Imager 680 (GE HealthCare).
TIFF files are available.

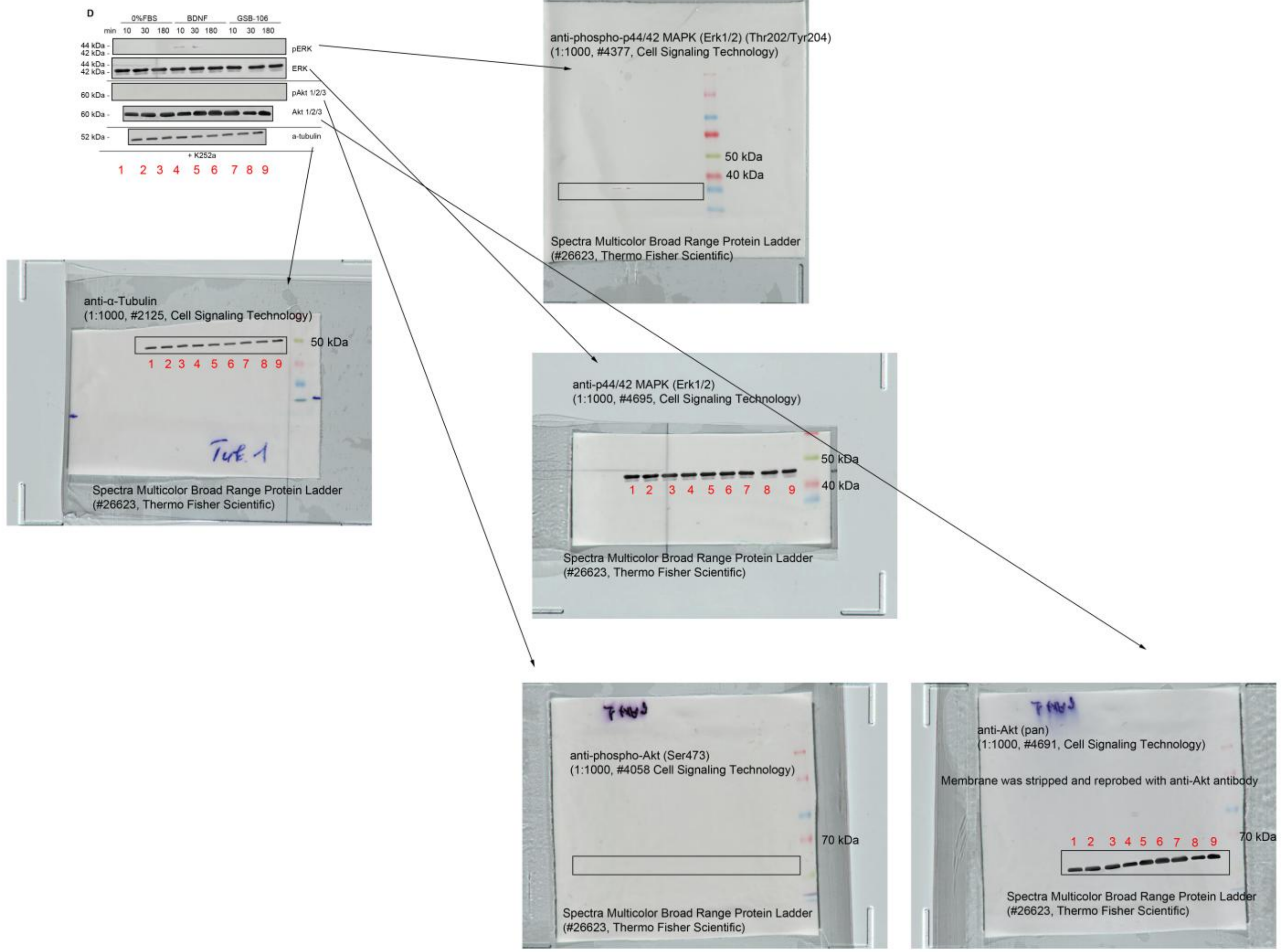

Zainullina et al., Figure 4

Original scans of blots, corresponding to figure 4E

Blots acquired on Amersham Imager 680 (GE HealthCare).
TIFF files are available.

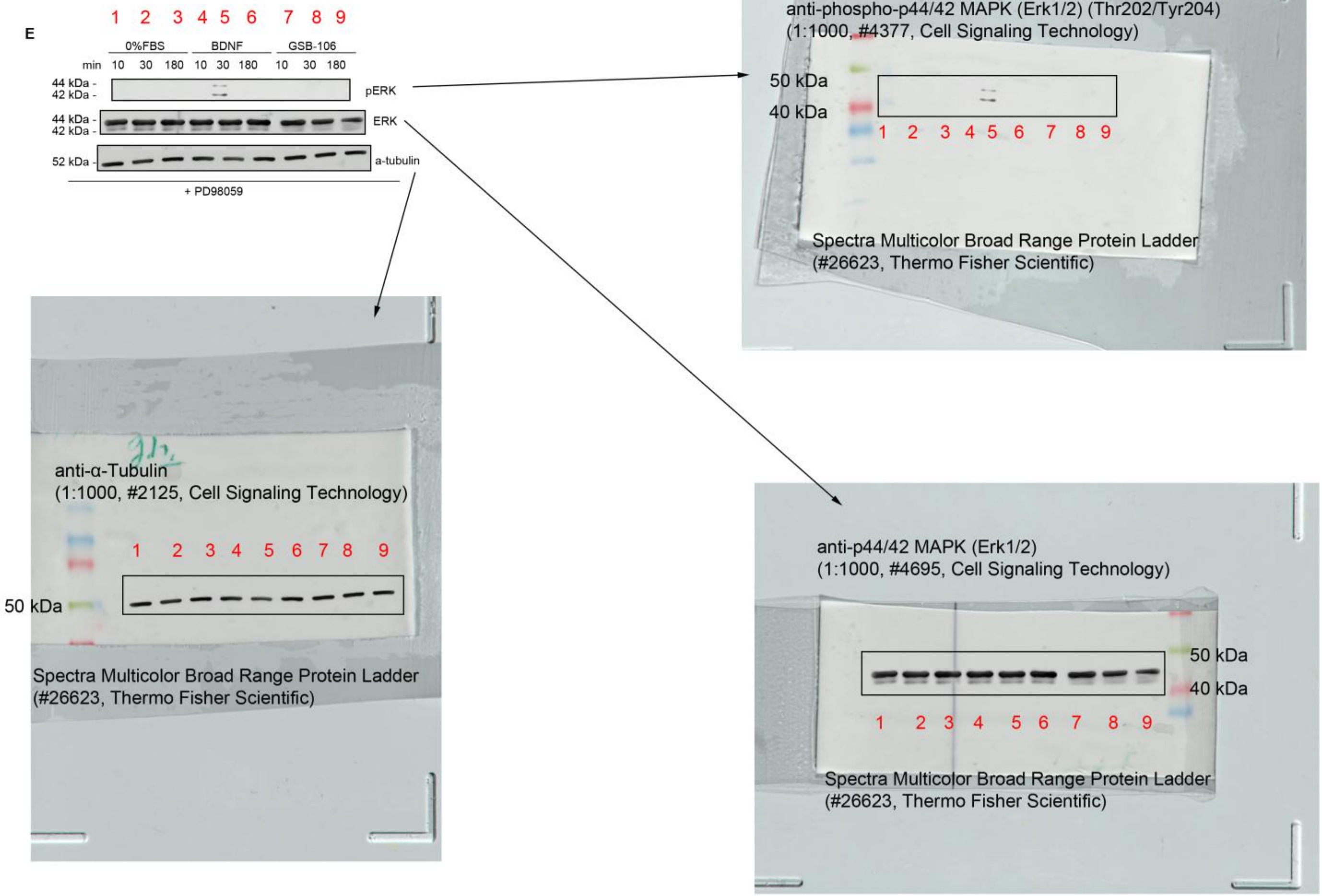

Zainullina et al., Figure 4

Original scans of blots, corresponding to figure 4F

Blots acquired on Amersham Imager 680 (GE HealthCare).
TIFF files are available.

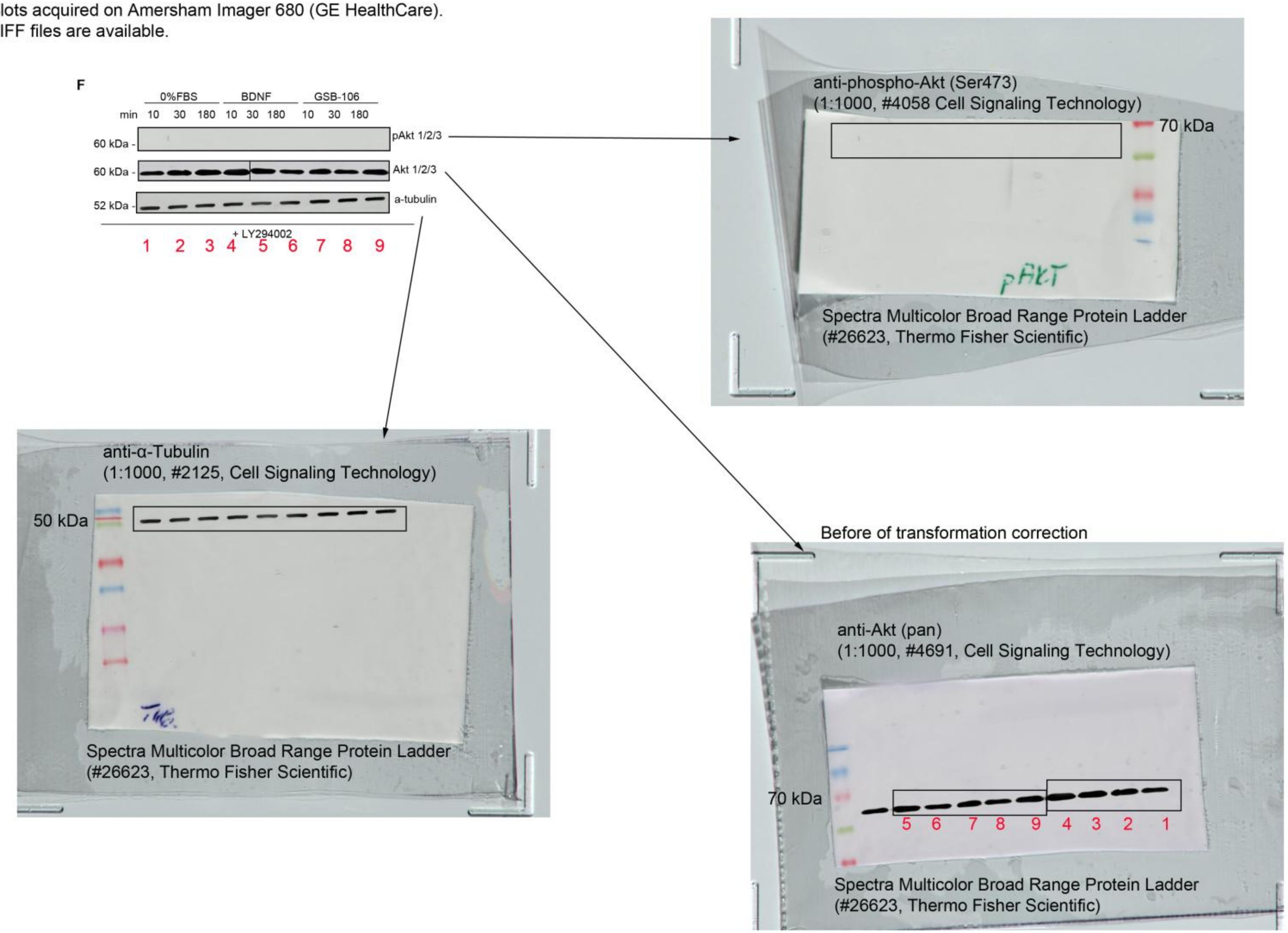

Zainullina et al., Figure 5 as submitted

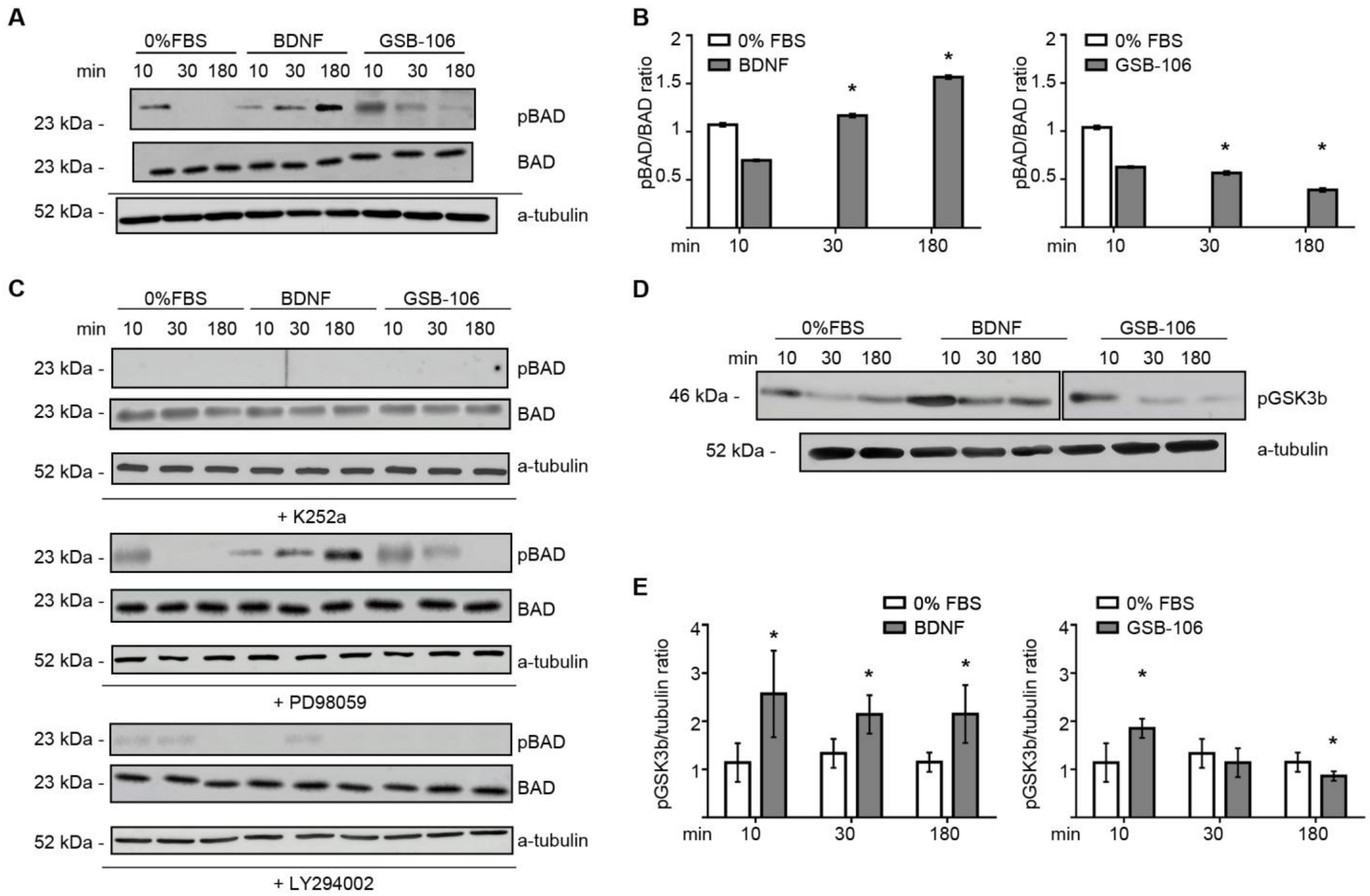

Zainullina et al., Figure 5

Original scans of blots, corresponding to figure 5A

Blots acquired on X-film. TIFF files are available.

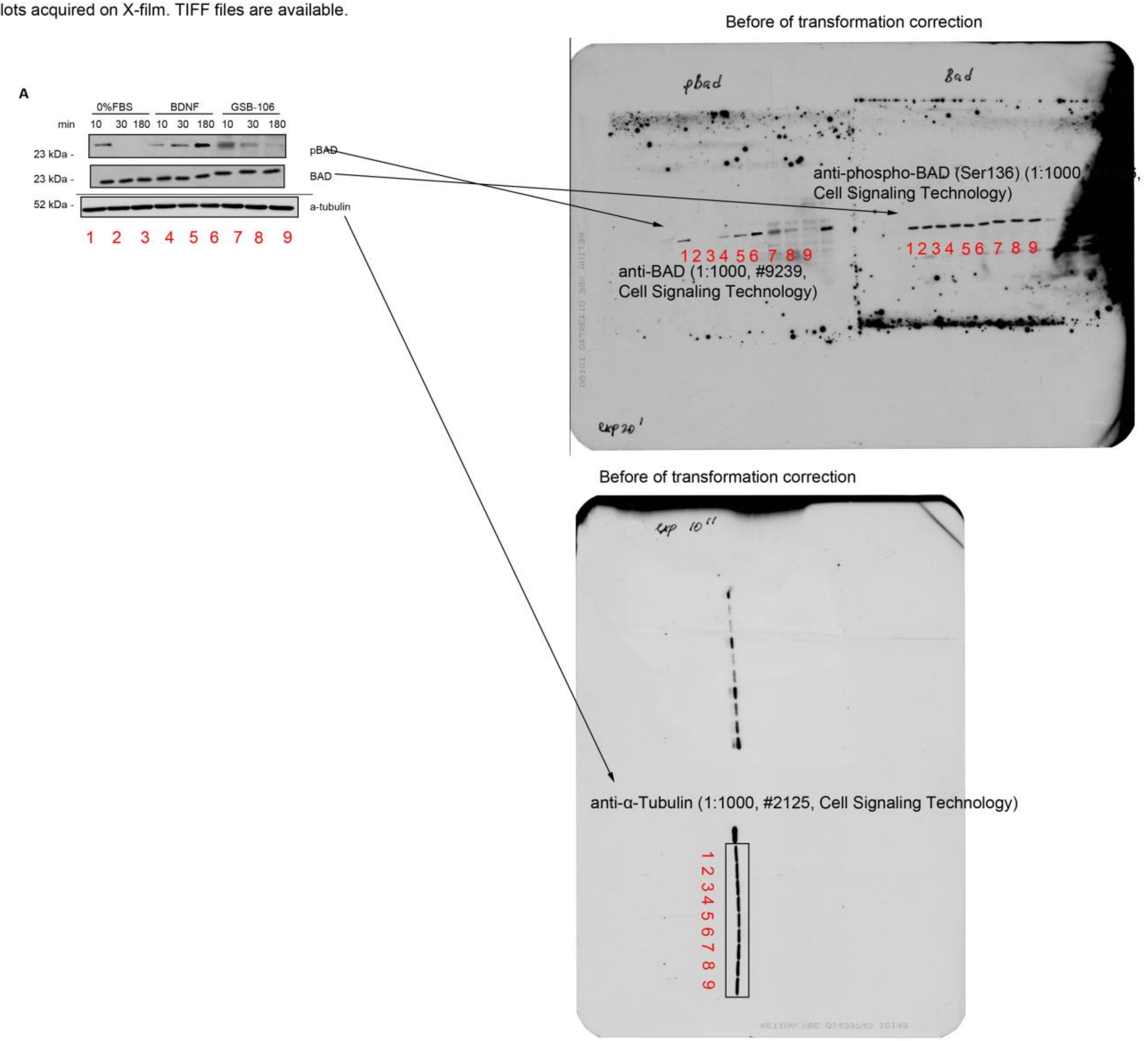

Zainullina et al., Figure 5

Original scans of blots, corresponding to figure 5C

Blots acquired on Amersham Imager 680 (GE HealthCare).
TIFF files are available.

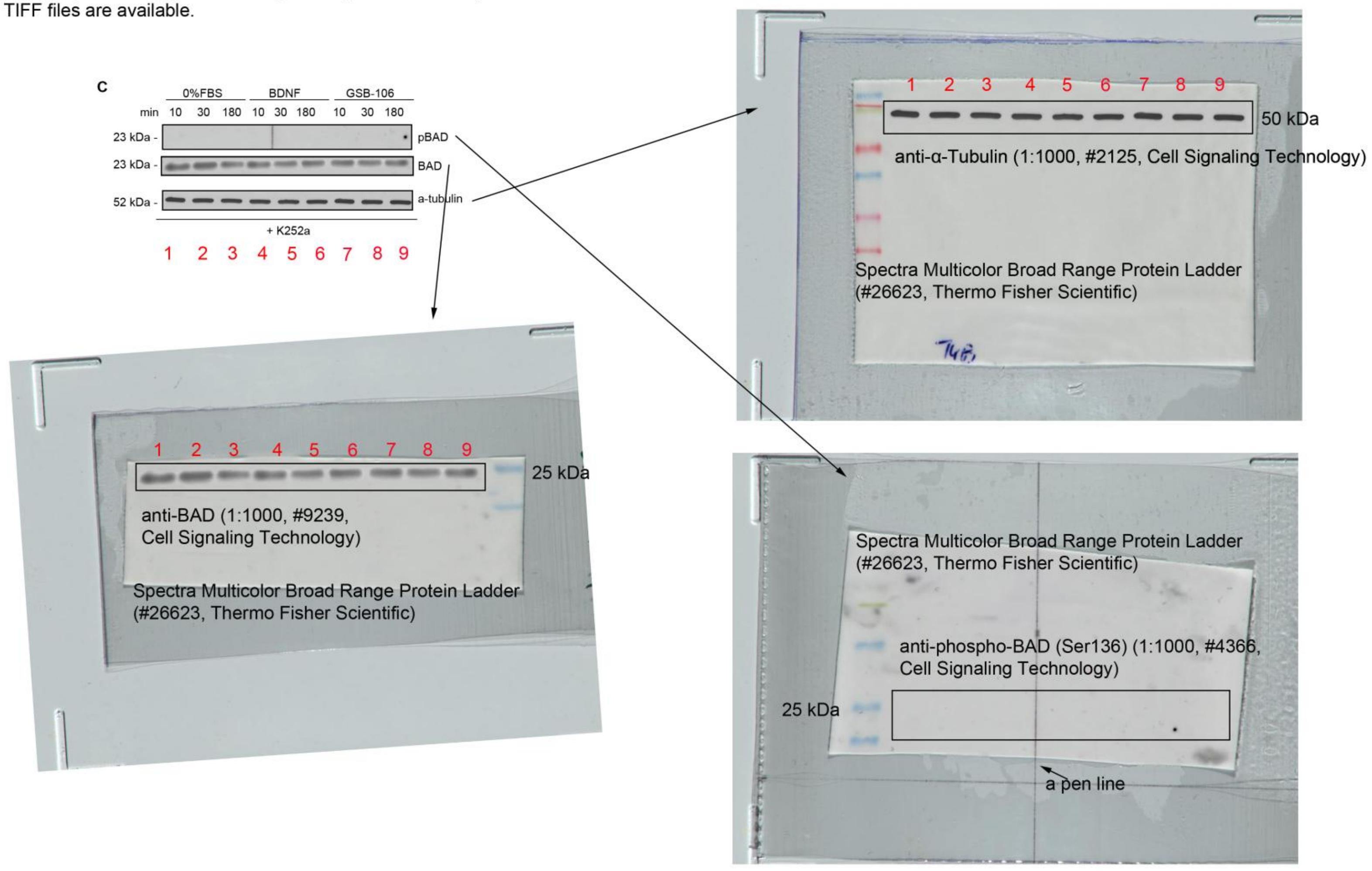

Zainullina et al., Figure 5

Original scans of blots, corresponding to figure 5C (continued)

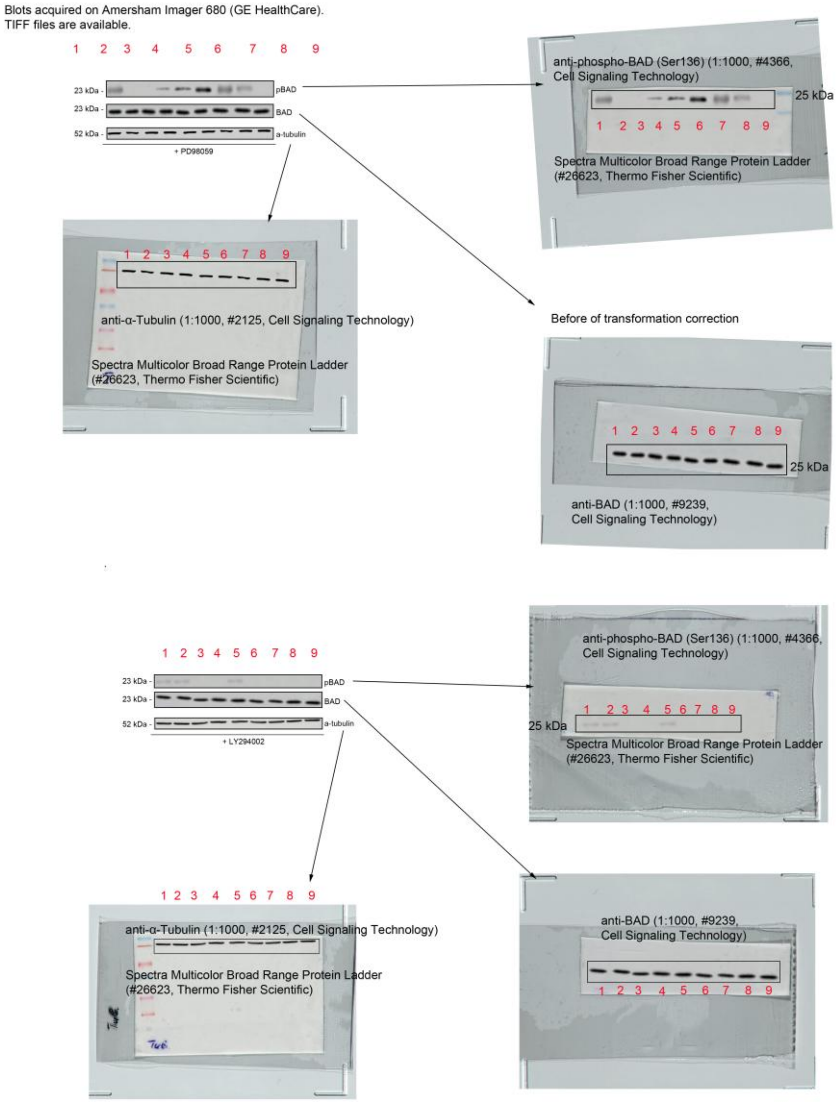

Zainullina et al., Figure 5

Original scans of blots, corresponding to figure 5D

Blots acquired on Amersham Imager 680 (GE HealthCare).
TIFF files are available.

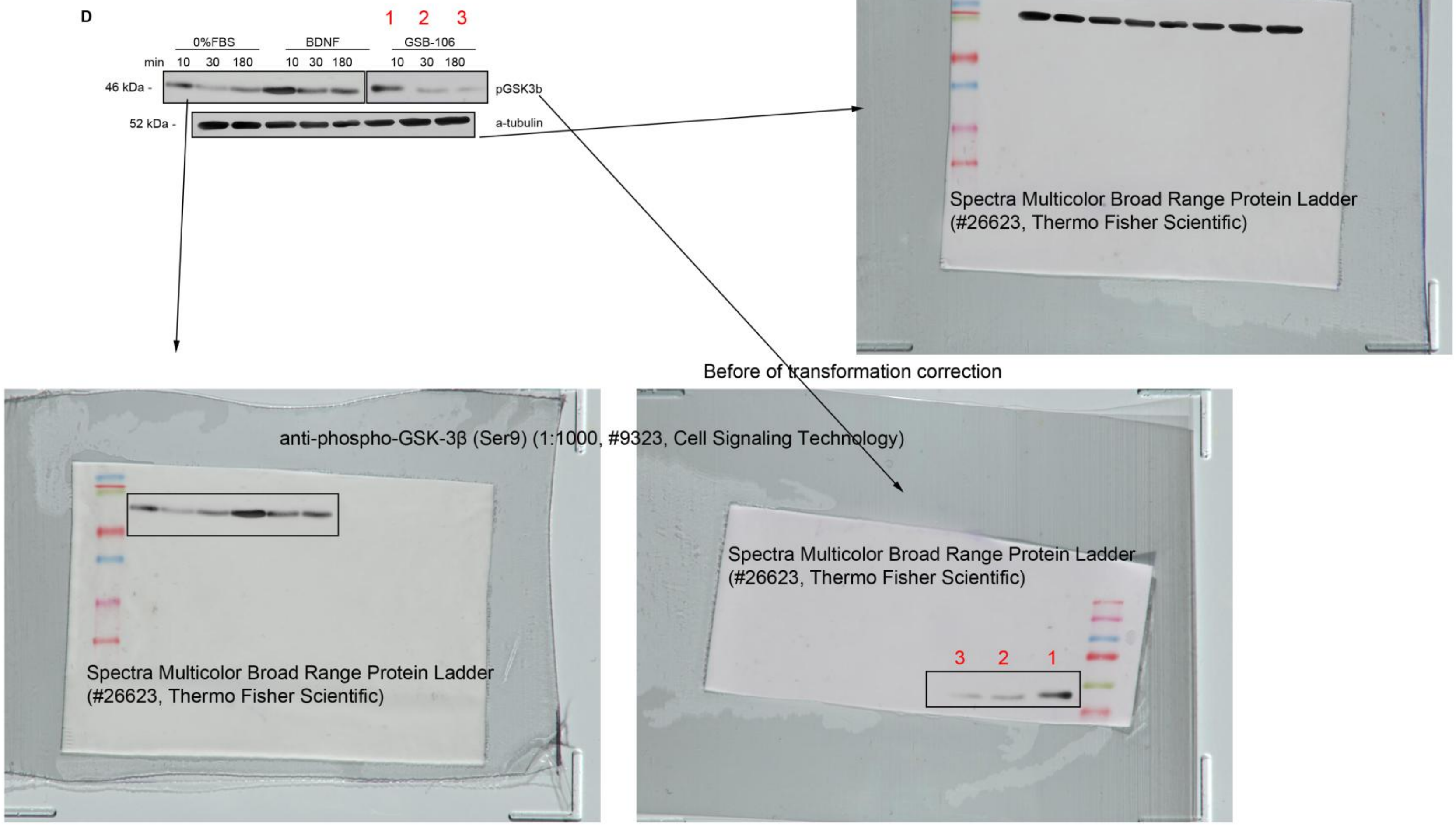

Zainullina et al., Figure 6 as submitted

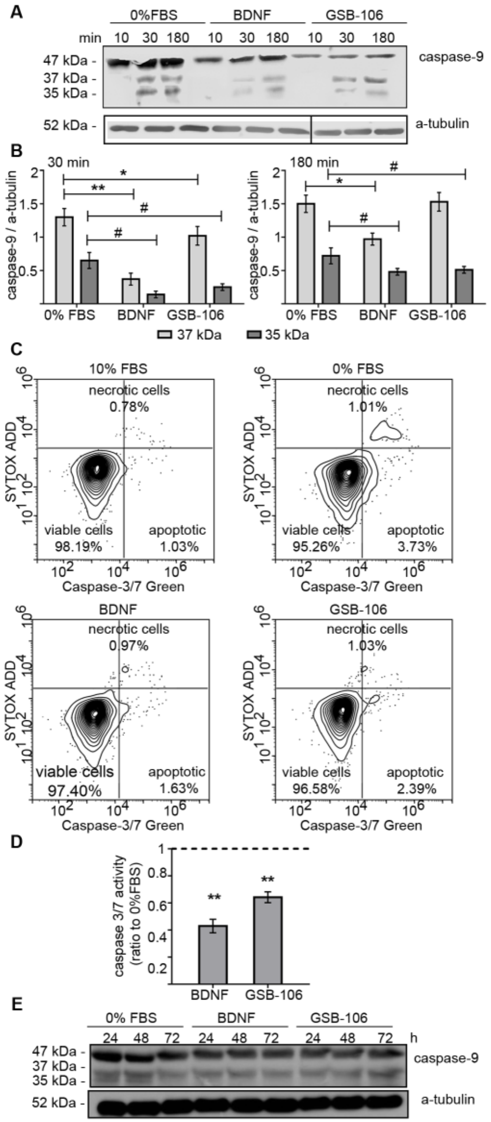

Zainullina et al., Figure 6

Original scans of blots, corresponding to figure 6A and 6E

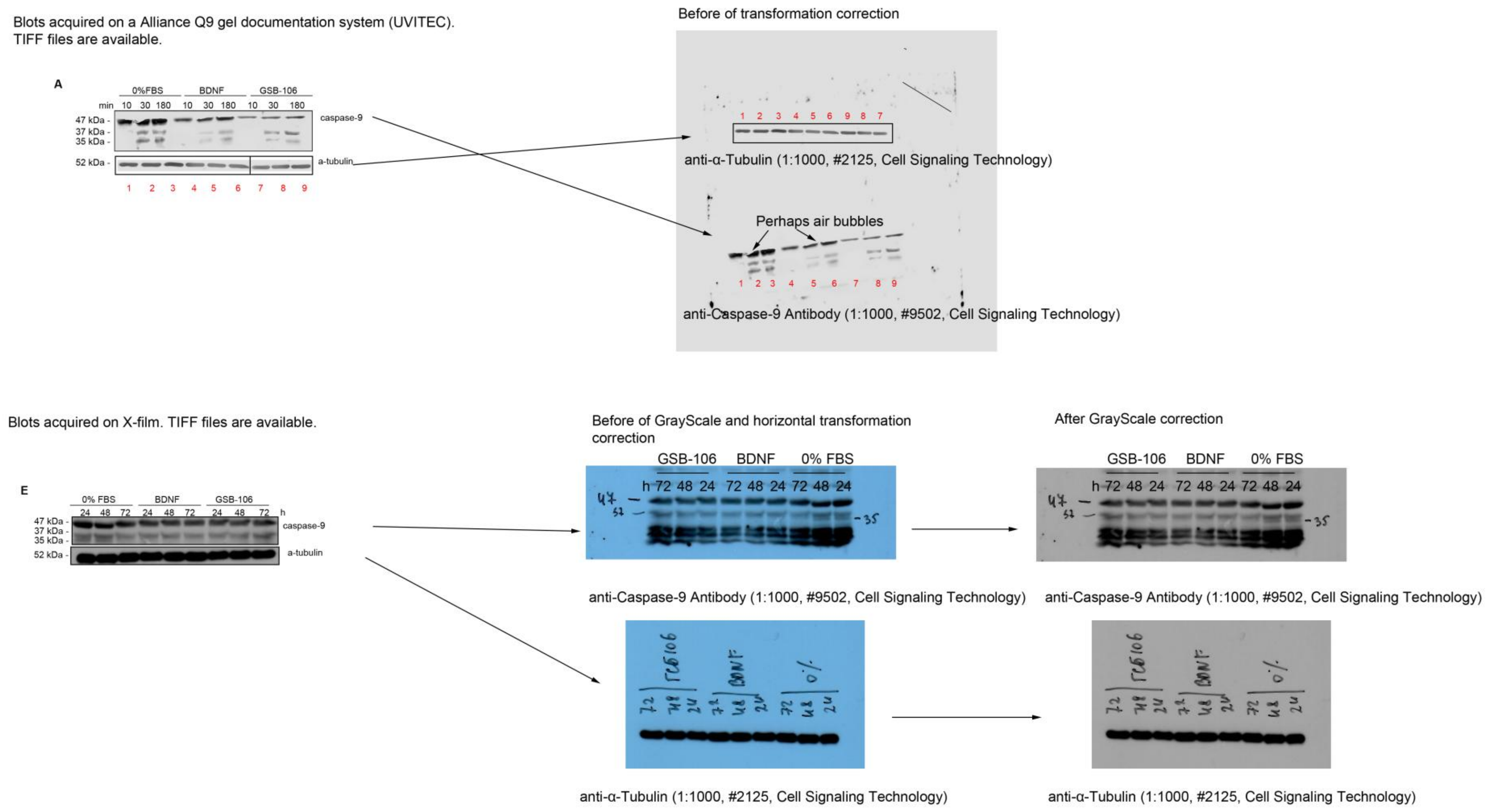

Zainullina et al., Supplementary Figure 1 as submitted

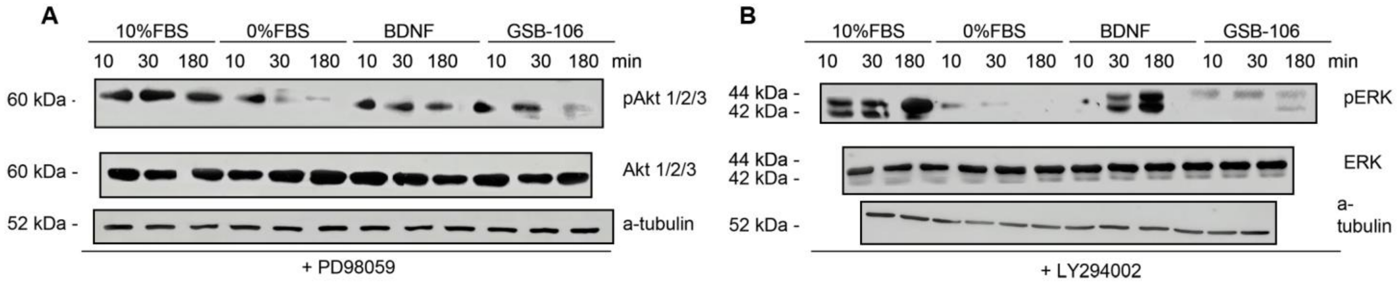

Zainullina et al., Supplementary Figure 1

Original scans of blots, corresponding to Supplementary Figure 1A and 1B

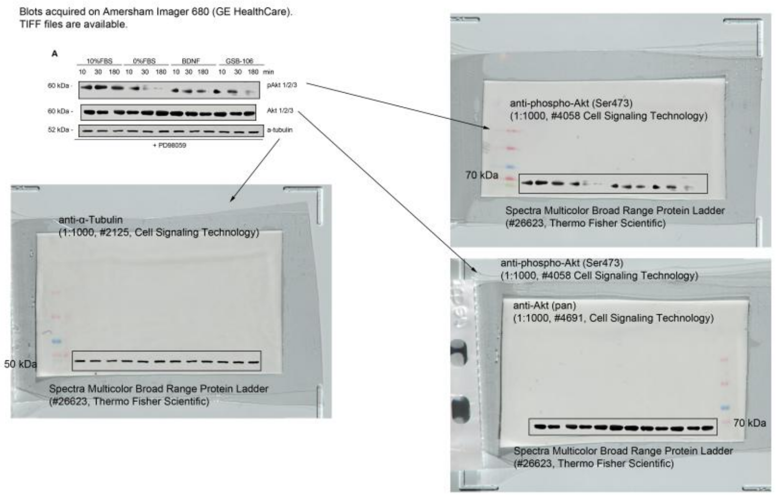

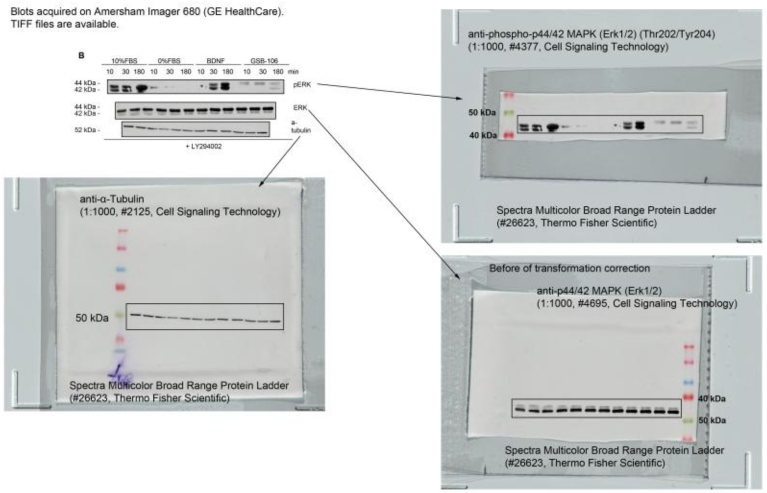

Supplement: Supplementary file 1 — Supplementary Information. [file 41598_2021_87435_MOESM1_ESM.pdf]
